# The generation of knock-in mice expressing fluorescently tagged galanin receptors 1 and 2

**DOI:** 10.1016/j.mcn.2015.08.006

**Published:** 2015-09

**Authors:** Niall Kerr, Fiona E. Holmes, Sally-Ann Hobson, Penny Vanderplank, Alan Leard, Nina Balthasar, David Wynick

**Affiliations:** a Schools of Physiology and Pharmacology and Clinical Sciences, Medical Sciences Building, University Walk, Bristol BS8 1TD, UK; b Wolfson Bioimaging Facility, Medical Sciences Building, University Walk, Bristol BS8 1TD, UK

**Keywords:** AOTF, acousto-optic tunable filter, BAC, bacterial artificial chromosome, CDS, coding sequence, CMV, cytomegalovirus, DOR, δ-opioid receptor, DR, dorsal raphe nucleus, DRG, dorsal root ganglia, EGFP, enhanced green fluorescent protein, ER, endoplasmic reticulum, ES cell, embryonic stem cell, FRT sites, FLP recombination target sites, GALP, galanin-like peptide, Gapdh, glyceraldehyde 3-phosphate dehydrogenase, GFP, green fluorescent protein, GPCRs, G protein-coupled receptors, hrGFP, humanized *Renilla* green fluorescent protein, ISH, in situ hybridization, LSN, lateral spinal nucleus, ME, median eminence, NPY, neuropeptide Y, nt, nucleotides, RNA-seq, next generation RNA sequencing, RT-PCR, reverse transcription polymerase chain reaction, TSA, tyramide signal amplification, UTR, untranslated region, uORFs, upstream open reading frames, GalR1, GalR2, Dorsal root ganglion, Spinal cord, Brain, uORF

## Abstract

The neuropeptide galanin has diverse roles in the central and peripheral nervous systems, by activating the G protein-coupled receptors Gal_1_, Gal_2_ and the less studied Gal_3_ (*GalR1*–*3* gene products). There is a wealth of data on expression of Gal_1–3_ at the mRNA level, but not at the protein level due to the lack of specificity of currently available antibodies. Here we report the generation of knock-in mice expressing Gal_1_ or Gal_2_ receptor fluorescently tagged at the C-terminus with, respectively, mCherry or hrGFP (humanized *Renilla* green fluorescent protein). In dorsal root ganglia (DRG) neurons expressing the highest levels of Gal_1_-mCherry, localization to the somatic cell membrane was detected by live-cell fluorescence and immunohistochemistry, and that fluorescence decreased upon addition of galanin. In spinal cord, abundant Gal_1_-mCherry immunoreactive processes were detected in the superficial layers of the dorsal horn, and highly expressing intrinsic neurons of the lamina III/IV border showed both somatic cell membrane localization and outward transport of receptor from the cell body, detected as puncta within cell processes. In brain, high levels of Gal_1_-mCherry immunofluorescence were detected within thalamus, hypothalamus and amygdala, with a high density of nerve endings in the external zone of the median eminence, and regions with lesser immunoreactivity included the dorsal raphe nucleus. Gal_2_-hrGFP mRNA was detected in DRG, but live-cell fluorescence was at the limits of detection, drawing attention to both the much lower mRNA expression than to Gal_1_ in mice and the previously unrecognized potential for translational control by upstream open reading frames (uORFs).

## Highlights

• We generated knock-in mice expressing fluorescently tagged galanin receptors 1 and 2.
• Gal_1_-mCherry fluorescence was associated with primary neuron somatic cell membrane.
• Gal_1_-mCherry protein expression in DRG, spinal cord and brain tissues is described.
• Gal_2_ mRNA expression is much lower than Gal_1_ in dorsal root ganglia.
• We propose that upstream open reading frames influence Gal_2_ protein expression.

## 1 Introduction

Galanin is a 29–30 amino acid neuropeptide that is dramatically induced after peripheral or central nervous system injury, and plays physiological roles in nociception, memory and cognition, anxiety-related behaviours, feeding, reproduction, neurite outgrowth and as a neuronal trophic factor (Cortes et al., 1990; Lang et al., 2015; Villar et al., 1989; Webling et al., 2012). The N-terminal 15 residues of galanin are strictly conserved between species and the N-terminal end is essential for biological activity (Lang et al., 2015; Webling et al., 2012), binding to the three galanin receptor subtypes Gal_1_, Gal_2_ and Gal_3_ (*GalR1*–*3* gene products), which are each Class A rhodopsin-like G protein-coupled receptors (GPCRs) that differ in sites of expression, functional coupling and signalling activities (Webling et al., 2012). The phenotypes of mice deficient in each of the galanin receptors have recently been reviewed (Brunner et al., 2014; Lang et al., 2015; Webling et al., 2012). Gal_1–3_ are also bound by galanin-like peptide (GALP), but not by the GALP alternatively spliced product alarin (Webling et al., 2012), and recently the neuropeptide spexin/NPQ (neuropeptide Q) was also reported to bind to Gal_2_ and Gal_3_ , but not to Gal_1_ (Kim et al., 2014).

In adult rat the expression of Gal_1_ mRNA is largely restricted to brain, spinal cord and dorsal root ganglia (DRG), whereas Gal_2_ mRNA is also detected in several peripheral tissues such as large intestine and uterus. In contrast, Gal_3_ mRNA has a more restricted distribution within brain, is rare in spinal cord and rare or not present in DRG, and expression in peripheral tissues is controversial (Burazin et al., 2000; Howard et al., 1997; O'Donnell et al., 1999; Shi et al., 2006; Waters and Krause, 2000; Webling et al., 2012). By in situ hybridization (ISH), Gal_1_ mRNA is more highly expressed than Gal_2_ overall in brain (Burazin et al., 2000) and by far the highest levels of Gal_2_ detected in the nervous system are in the DRG (O'Donnell et al., 1999).

In adult mouse brain the distribution of Gal_1_ mRNA by ISH is largely similar to rat (Hohmann et al., 2003), whereas the absence of specific [^125^I]-galanin binding sites in any region of adult Gal_1_-deficient brain (Jungnickel and Gundlach, 2005) suggests a species-specific, greatly reduced expression of Gal_2_ mRNA. However, it is still detectable by the more sensitive RT-PCR method in both mouse whole brain (Hobson et al., 2006; Jacoby et al., 2002) and subregions including the amygdala, hippocampus and hypothalamus (Brunner et al., 2014; Hawes et al., 2005; He et al., 2005; Shi et al., 2006; Zhao et al., 2013), as well as in spinal cord (Jacoby et al., 2002), DRG, trigeminal and nodose sensory ganglia (Hobson et al., 2006; Page et al., 2007) and several peripheral tissues (Barreto et al., 2011; Hobson et al., 2006; Jacoby et al., 2002; Kim and Park, 2010; Pang et al., 1998). Less work has been reported on Gal_3_ mRNA expression, but by RT-PCR it is detected in mouse whole brain and some subregions (Brunner et al., 2014; Hawes et al., 2005; Zhao et al., 2013), nodose ganglion (Page et al., 2007) and several peripheral tissues (Barreto et al., 2011; Brunner et al., 2014; Kim and Park, 2010), but is at the limits of detection in both spinal cord and DRG (Hobson et al., 2006; Jacoby et al., 2002).

Current antibodies against Gal_1_ or Gal_2_ are non-selective under standard immunodetection conditions, with identical immunoreactivity patterns in wild-type and receptor knockout mice (Hawes and Picciotto, 2005; Lang et al., 2015; Lu and Bartfai, 2009; F.E.H., P.V. and D.W., unpublished). To delineate the expression of Gal_1_ and Gal_2_ at the protein level we wished to tag each receptor with fluorochromes. The C-terminal tagging of GPCRs with green fluorescent protein (GFP) is generally thought to have no significant effect on GPCR properties e.g. ligand binding, signal transduction and intracellular trafficking (Ceredig and Massotte, 2014), and both Gal_1_ and Gal_2_ have been shown to be functional when C-terminally tagged with enhanced GFP (EGFP) or its variants and expressed in cell lines (Wirz et al., 2005; Xia et al., 2004, 2008). The GPCR superfamily is the largest group of cell surface receptors and are the targets of around one third of marketed drugs, yet to date the only knock-in mice that express a fluorescently-tagged GPCR are Rhodopsin-EGFP and two rhodopsin mutant variants, δ-opioid receptor (DOR)-EGFP and the recently reported μ-opioid receptor (MOR)-mCherry (Ceredig and Massotte, 2014; Erbs et al., 2015; Scherrer et al., 2006). Transgenic mice have been successfully generated expressing either humanized *Renilla* GFP (hrGFP; Stratagene-Agilent; Zeng et al., 2003) under the control of various endogenous promoters (Sakata et al., 2009; van den Pol et al., 2009; Voigt et al., 2012), or with widespread expression of the monomeric red fluorescent protein mCherry under the control of a ubiquitin-C promoter (Fink et al., 2010; Shaner et al., 2004). Here we describe the generation and initial characterization of Gal_1_-mCherry and Gal_2_-hrGFP knock-in mice, focussing on expression in DRG, spinal cord and brain.

## 2 Materials and methods

### 2.1 DNA sequence analysis

Planning for knock-in gene characterizations used mouse reference genome Build 37.1, vector hrGFP-FRT*neo*FRT (see below, Section 2.2) and mCherry cDNA (AY678264) sequences. The RepeatMasker 3.2.9 programme (Smit, Hubley and Green, 1996–2010, http://www.repeatmasker.org) was used to select regions of the *GalR1* or *GalR2* genes for retrieval target sites or Southern probes that avoided repetitive DNA elements, and homology arm targets that minimized the presence of repetitive DNA elements. Ribosome profiling data (Ingolia et al., 2011) was accessed using the GWIPS-viz. browser (http://gwips.ucc.ie; Michel et al., 2014). The ribosome density peaks for the uORF6 and *GalR2* initiation codons were at, respectively, Chr11 nucleotides (nt) 116,281,254–286 and 116,281,474–509 of the GRCm38/mm10 genome assembly (GWIPS-viz. ribo-seq coverage plot; Ingolia et al., 2011; Michel et al., 2014).

### 2.2 Generation of GalR1-mCherry-[neo^+^] and GalR2-hrGFP-[neo^+^] knock-in mice

**Fig. 1:**
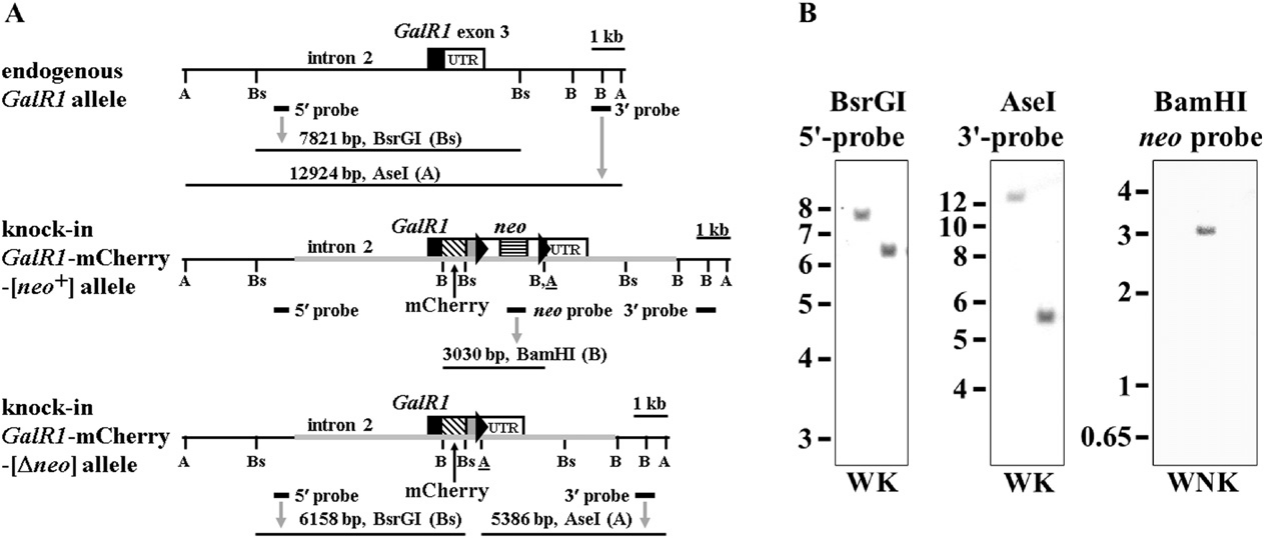
Gene-targeting of *GalR1* and genotype analysis. (A) Schematic diagram of endogenous *GalR1* (*top panel*), knock-in *GalR1*-mCherry-[*neo*^+^] (*middle panel*) and knock-in *GalR1*-mCherry-[Δ*neo*] (*bottom panel*) alleles. Indicated relative locations are *GalR1* exon 3 with coding sequence (CDS; black filled box) and endogenous 3′-UTR (‘UTR’; NM_008082, not full-length), and restriction sites AseI (A), BsrGI (Bs) and BamHI (B). The knock-in *GalR1*-mCherry-[*neo*^+^] DNA (*middle panel*) includes the following elements: the targeting construct (grey horizontal thickened line); the 3′ end of the *GalR1* CDS joined in-frame by a 21 nucleotide linker, encoding the flexible linker RDPPVAT (Gibbs et al., 2003; Lobbestael et al., 2010), to mCherry CDS (diagonal banded box; mCherry protein sequence AAV52164); a heterologous 3′-UTR including the SV40 early region poly(A) site (grey filled box); downstream FRT sites (right arrowheads) flanking a SV40-*neo* cassette selection marker with a HSV (herpes simplex virus) thymidine kinase (*TK*) poly(A) site; an introduced AseI restriction site (A) immediately downstream of the 3′ FRT site; and the endogenous 3′-UTR (‘UTR’) (see Materials and methods section). Locations of external 5′ and 3′ probes, *neo* probe, and hybridizing DNA fragments are also shown (see Supplementary Fig. 1A for more detailed image of [*neo*^+^] allele). Note that in the *GalR1*-mCherry-[Δ*neo*] knock-in allele (*bottom panel*) the SV40-*neo* cassette flanked by FRT sites has been removed by FLPe. (B) Southern blot analysis validates correct insertion of the targeting construct. Wild-type (W) and *GalR1*-mCherry-[Δ*neo*] knock-in line 33 (K) mouse tail DNA were digested and hybridized with: BsrGI and *GalR1* 5′ external probe (*left panel*; W: 7821 bp, K: 6158 bp); AseI and *GalR1* 3′ external probe (*middle panel*; W: 12,924 bp, K: 5386 bp); and BamHI and *neo* probe, which only hybridized to positive-control heterozygous *GalR1*-mCherry-[*neo*^+^] ES cell clone 33 (*right panel*; lane N: 3030 bp). The relative distance travelled by DNA ladder fragments (1 kb Plus, Life Technologies) is indicated in kb on the left of each panel.

**Fig. 2:**
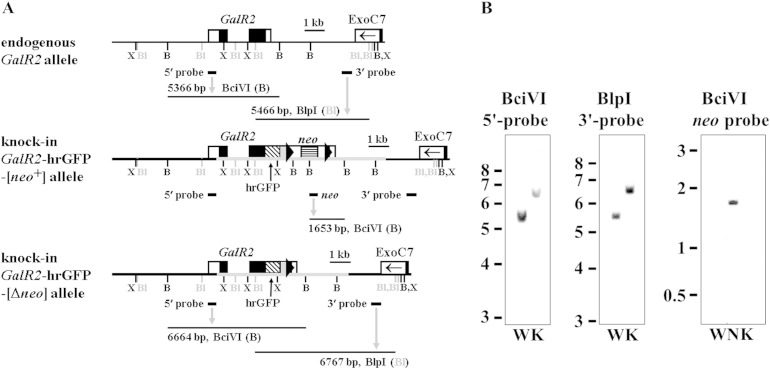
Gene-targeting of *GalR2* and genotype analysis. (A) Schematic diagram of endogenous *GalR2* (*top panel*), knock-in *GalR2*-hrGFP-[*neo*^+^] (*middle panel*) and knock-in *GalR2*-hrGFP-[Δ*neo*] (*bottom panel*) alleles. Indicated relative locations are the two exons of *GalR2* (boxed) with coding sequences (CDS; black filled boxes); the downstream terminal exon of *ExoC7* (exocyst complex component 7) on the other DNA strand (box with arrowhead); and restriction sites XmnI (X), BlpI (Bl, greytone) and BciVI (B). [XmnI sites are shown as used to characterize the [*neo*^+^] allele (Supplementary Fig. 2).] The knock-in *GalR2*-hrGFP-[*neo*^+^] DNA (*middle panel*) includes the targeting construct (grey horizontal thickened line), and the 3′ end of the *GalR2* CDS (exon 2) joined to knock-in elements as in Fig. 1A except for exchanging hrGFP (diagonal banded box; hrGFP protein sequence AAK63811) for mCherry. Locations of external 5′ and 3′ probes, *neo* probe, and hybridizing DNA fragments are also shown (see Supplementary Fig. 2A for more detailed image of [*neo*^+^] allele). Note that in the *GalR2*-hrGFP-[Δ*neo*] knock-in allele (*bottom panel*) the SV40-*neo* cassette flanked by FRT sites has been removed by FLPe. (B) Southern blot analysis of tail DNA from wild-type (W) and *GalR2*-hrGFP-[Δ*neo*] knock-in line 32 (K) mice validates correct insertion of the targeting constructs. DNA was digested and hybridized with: BciVI and *GalR2* 5′ external probe (*left panel*; W: 5366 bp, K: 6664 bp); BlpI and *GalR2* 3′ external probe (*middle panel*; W: 5466 bp, K: 6767 bp); and BciVI and *neo* probe, which only hybridized to positive-control heterozygous *GalR2*-hrGFP-[*neo*^+^] ES cell clone 32 (*right panel*; lane N: 1653 bp). DNA ladder as Fig. 1B.

Mouse genomic clones including either *GalR1* or *GalR2* genes from the bMQ mouse strain 129S7 (129Sv) bacterial artificial chromosome (BAC) library (inserts 89–178 kb; Source BioScience; Adams et al., 2005) were electroporated into strain EL250 *Escherichia coli* (Lee et al., 2001). This allowed temperature-inducible, lambda Red-mediated, homologous recombination into the BAC (Copeland et al., 2001; Lee et al., 2001) of PCR products from either vector hrGFP-FRT*neo*FRT (Balthasar et al., 2004; Parton et al., 2007; hrGFP derived from Stratagene-Agilent vector phrGFP-1, Zeng et al., 2003; Fig. 2A, *middle panel*) flanked by *GalR2* homologous sequence or vector mCherry-FRT*neo*FRT (hrGFP exchanged for mCherry; Shaner et al., 2004) flanked by *GalR1* homologous sequence. Within the latter PCR product an AseI restriction site was introduced immediately downstream of the 3′ FRT site (Fig. 1A, *middle panel*), for use in Southern blot digests, and the mCherry/hrGFP heterologous 3′-untranslated region (UTR) has identity to nt 705–1193 of vector pCMV-Script (AF028239) which includes the SV40 early region poly(A) site (Connelly and Manley, 1988; J02400, nt 2828–2547). Correct insertion into the BAC was validated by DNA sequencing of cloned PCR products of each junction. All DNA sequencing was by Source BioScience, Oxford.

Retrieval from the recombined BAC clones by gap repair (Copeland et al., 2001; Lee et al., 2001) into PCR-amplified vector pCR-Blunt (Invitrogen) was mediated by either *GalR1* target sequences with adjacent introduced rare SwaI restriction sites, or *GalR2* target sequences with adjacent introduced BstZ17I restriction sites. Recombined plasmid DNAs were transformed into STBL3 *E. coli* (Invitrogen) and the final targeting constructs were excised with either SwaI (*GalR1*-mCherry-FRT*neo*FRT; 11.3 kb, with *GalR1* homology arms of 4.2 and 3.9 kb) or BstZ17I (*GalR2*-hrGFP-FRT*neo*FRT; 8.1 kb, with *GalR2* homology arms of 2.3 and 2.7 kb).

Targeting construct inserts were electroporated into embryonic stem (ES) cell line E14.1a (strain 129P2/OlaHsd; Downing and Battey, 2004; Hooper et al., 1987) and the SV40-*neo* cassette selected with 250 μg/ml G418 by Geneta (Dept. of Biochemistry, University of Leicester). G418-resistant ES cell clones were screened for correct targeting by PCR (data not shown) and real-time quantitative genomic PCR, and expanded clones were screened by Southern blot analysis (Supplementary Materials and Methods). Selected clones were karyotyped to confirm euploidy, and three ES cell clones of each knock-in gene were injected into 3.5 day old blastocysts from C57BL/6J mice to produce chimeric mice (Geneta). These were crossed to strain 129P2/OlaHsd mice and germline transmission was assessed by PCR genotyping (see below).

### 2.3 Animals

Mice were housed in a temperature- and humidity-controlled colony on a 14:10 h light–dark cycle, and fed standard chow and water ad libitum. Procedures were carried out in accordance with the U.K. Animals (Scientific Procedures) Act, 1986 and associated guidelines. Ear-punch biopsies were used for PCR genotyping, animals were killed by cervical dislocation to obtain DRG for RT-PCR analysis (Section 2.6), or primary DRG cultures (Section 2.7). Three mice had peripheral transection of the right sciatic nerve prior to perfusion seven days later to obtain ipsilateral (axotomized) lumbar L4 and L5 DRG (Holmes et al., 2008) for immunohistochemistry (Section 2.9).

### 2.4 PCR genotyping to distinguish knock-in from endogenous receptor alleles

Mouse PCR genotyping for *GalR1* used the three primers 5′-AGCCTTCCCACTGACGCCAGCTT-3′, 5′-CAGAACTTACTTTACACCATGGAGATC-3′ and 5′-TCGAACTCGTGGCCGTTCACGGA-3′, which amplified an endogenous *GalR1* product of 547 bp and a *GalR1*-mCherry knock-in product of 348 bp. PCR genotyping for *GalR2* used primers 5′-CGAGGAGAGCTTCAGGCCGAGT-3′, 5′-CACCCTGTAAAGTCCCAGAGACGT-3′ and 5′-CTGGTTGCCGAACAGGATGTTGC-3′, which amplified an endogenous *GalR2* product of 543 bp and a *GalR2*-hrGFP knock-in product of 308 bp. PCRs with FastStart Taq DNA polymerase (Roche) and HPLC-purified primers (MWG Eurofins) used cycling conditions of 94 °C for 7 min, 40 cycles of [94 °C, 30 s; 63 °C, 45 s; 72 °C, 45 s], and a final 72 °C incubation for 10 min.

### 2.5 Deletion of the FRT-flanked SV40-neo cassette from knock-in mice

Homozygous strain C57BL/6J *ACTB*:*FLPe* transgenic mice (Buchholz et al., 1998; Rodriguez et al., 2000) from the Bristol University colony (provided by Dr. Alastair Poole and N.B.) were PCR genotyped using primers 5′-CAATACCTGATCACTACTTCGCACT-3′ and 5′-CATGTCTGATCCTCGAGGAGCTC-3′ under standard cycling conditions (Section 2.4), which amplified the expected 362 bp sequenced product.

Heterozygous *GalR1*-mCherry-[*neo*^+^] knock-in mice were crossed to *ACTB*:*FLPe* transgenic mice, and offspring both heterozygous for *GalR1*-mCherry and genetically mosaic for deletion of the SV40-*neo* cassette (Δ*neo*) by PCR genotyping (i.e. both Δ*neo* and *neo*^+^) were then crossed to strain 129P2/OlaHsd. Offspring were PCR genotyped in separate reactions for *GalR1*-mCherry heterozygosity (Section 2.4), presence of *neo* and presence of Δ*neo*. Primers for presence of *neo* were 5′-GCATACGCTTGATCCGGCTACCT-3′ and 5′-CTCCTTCCGTGTTTCAGTTAGCCT-3 (630 bp product), and for presence of Δ*neo* were 5′-GTCTGTTCATGATCATAATCAGCCAT-3′ (UTR-F; heterologous 3′-UTR sequence) and 5′-CAGAACTTACTTTACACCATGGAGATC-3′ (GalR1R) which amplified the expected 626 bp sequenced product. A similar breeding strategy was used with the heterozygous *GalR2*-hrGFP-[*neo*^+^] knock-in mice, except that primers for presence of Δ*neo* were UTR-F and 5′-CCTCAAACTTGATGGCTGGCTTTG-3′ (GalR2R) which with annealing at 65 °C amplified the expected 528 bp sequenced product.

Frozen sperm from homozygous *GalR1*-mCherry (line 33) or *GalR2*-hrGFP (line 32) knock-in mice were deposited at the MRC Frozen Embryo and Sperm Archive and are available from https://www.infrafrontier.eu/(GalR1-mCherry, EMMA stock ID EM:08192; GalR2-hrGFP, EMMA stock ID EM:08193).

### 2.6 Real-time quantitative RT-PCR

Lumbar DRG from individual, age-matched 7–8 week old wild-type, *GalR1*-mCherry or *GalR2*-hrGFP mice (each *n* = 5; 1 male, 4 females) were frozen on dry ice and stored at − 80 °C, prior to total RNA isolation and reactions containing reverse transcriptase (RT +) or without enzyme (RT − control) (Kerr et al., 2004). Real-time quantitative RT-PCR (reverse transcription polymerase chain reaction) assays and primer and probe sets (Applied Biosystems) for Gal_1_, Gal_2_, galanin and endogenous control glyceraldehyde 3-phosphate dehydrogenase (Gapdh) were as reported (Hobson et al., 2006), except for the corrected Gal_2_ probe sequence 5′-TTCCTCACTATGCACGCCAGCAGC-3′ (mGalR2-46TAQ) used then and herein. Relative mRNA expression levels were derived by the comparative threshold cycle (C_t_) method (2^− ΔΔCT^) normalized to Gapdh (Hobson et al., 2006), with results presented as mean of log transformed data plus SEM. Sample identities were confirmed by standard RT-PCR for Gal_1_-mCherry and Gal_2_-hrGFP receptor-fluorescent tag products (data not shown).

### 2.7 DRG culture and live-cell imaging

DRG cultures from 8 week old mice (Hobson et al., 2013) were generated from individual animals, and neurons were replated onto glass-bottom microwell dishes (MatTek) treated with 0.5 mg/ml polyornithine and 5 μg/ml laminin (Sigma). Immediately prior to imaging, medium was changed to air-buffered L15 (Sigma) supplemented with 5% horse serum, 1 mM l-glutamine and 10 ng/ml gentamicin. Cell images were recorded using a Leica TCS SP8 confocal system with enhanced sensitivity due to the GaAsP Hybrid detector (HyD), attached to a Leica DMI6000 inverted epifluorescence microscope with a 63 ×/1.30 Glycerol objective lens (Leica Microsystems), at 37 °C. Imaging parameters were selected to optimize confocal resolution. Specifically, GalR1-mCherry was detected by excitation with a HeNe 594 nm laser (Anderson et al., 2006; 80% acousto-optic tunable filter, AOTF) and emission detected at 600–660 nm with 330% gain, 6 × line accumulation, and 2 × frame averaging. GalR2-hrGFP was detected by excitation with a 488 nm argon laser (35% power, with 70% AOTF) and emission detected at 492–538 nm with 400% gain, 6 × line accumulation, and 2 × frame averaging. Confocal images were detected as *z* stacks of *x*–*y* images taken at 1 μm intervals, and were acquired using LCS (Leica) software. Images designated ‘adjusted’ used linear brightness/contrast functions of Adobe Photoshop software.

### 2.8 Receptor internalization studies and quantification of somatic cell membrane associated fluorescence

Immediately prior to imaging the medium was changed to FluoroBrite DMEM (Life Technologies) to decrease background fluorescence, with medium supplements B-27 (Life Technologies), 1 mM l-glutamine and 10 ng/ml gentamicin. Imaging was at 37 °C with 5% CO_2_ enrichment. Single focal planes were imaged to reduce potential photo-bleaching, with 6 × line accumulation and without frame averaging. Porcine galanin (1–29) was from Bachem. Quantification of somatic cell membrane fluorescence (Scherrer et al., 2006) of GalR1-mCherry from original confocal images used Volocity software (Perkin Elmer) to define both the area within the cell membrane (intracellular fluorescence) and between this and the outside of the somatic cell membrane (surface fluorescence). Results are presented as mean plus SEM.

### 2.9 Immunohistochemical staining

Mice were deeply anaesthetised and transcardially perfused with PBS followed by 4% paraformaldehyde/PBS. Brains, lumbar spinal cords and lumbar L4 and L5 DRGs were dissected and post-fixed for 24 h in 4% paraformaldehyde/PBS then transferred to 20% sucrose/PBS for 24 h at 4 °C. Spinal cords and DRG were placed in OCT embedding matrix (CellPath) and frozen on dry ice, and brains were frozen on dry ice. Tissue was stored at − 80 °C until use. 10 μm sections of DRG and 30 μm sections of spinal cord and brain were cut on a cryostat. DRG were collected directly onto Polysine slides (Thermo Scientific), or after immunohistochemical processing of floating sections for brain and cord. mCherry was detected using the TSA™ (tyramide signal amplification) Plus Fluorescein System (PerkinElmer). Sections were processed at room temperature as follows: incubated in 0.3% H_2_O_2_/PBS for 30 min to quench endogenous peroxidase; washed 1 × 5 min with TN (0.1 M Tris–HCl, pH 7.5; 0.15 M NaCl); blocked for 30 min in TNB (0.5% Blocking Reagent in TN); and incubated for 18 h in rabbit anti-DsRed antibody (*Discosoma* red fluorescent protein; Erbs et al., 2015; Voigt et al., 2012; Clontech cat. no. 632,496) diluted 1:500 in TNB. Sections were washed 3 × 5 min in TN; incubated in HRP anti-rabbit IgG (Vector Laboratories cat. no. PI-1000) diluted 1:200 in TNB for 1 h; washed 3 × 5 min in TN; incubated in fluorescein-conjugated tyramide (TSA™ Plus Fluorescein System; PerkinElmer) at 1:50 for 10 min. Sections were washed 3 × 10 min in TN, floating sections collected onto slides, then all sections mounted in Vectashield (Vector Laboratories) and coverslipped. Immunohistochemistry of wild-type tissue verified the specificity of the primary antibody, and omission of the primary antibody verified the absence of non-specific staining by the secondary antibody.

The polyclonal anti-hrGFP antibody (Sakata et al., 2009; Zhang et al., 2013; Stratagene-Agilent 240,142) was used as above diluted at 1:500 or 1:5000 on DRG (± axotomy) and brain, and at 1:500–1:30,000 on spinal cord (± axotomy), each with wild-type control tissue.

Gal_1_-mCherry immunofluorescence was detected by excitation with a 488 nm argon laser and emission detected at 492–538 nm on a Leica TCS SP5-II confocal system attached to a Leica DMI6000 inverted epifluorescence microscope with dry 20 × or oil 40 × objective lens. Confocal images were detected as *z* stacks of *x*–*y* images taken at 1 μm (DRG/brain) or 2 μm (cord, unless stated in Fig. 6N and O) intervals with 4 × line accumulation, and selected images extracted using Volocity software (Perkin Elmer). Photomicrographs of Gal_1_-mCherry immunofluorescence in brain at lower magnifications (2.5 ×, 5 ×) used IM50 Image Manager software (Leica; Holmes et al., 2008) with gain set at 1 (Fig. 7), as constrained by high immunoreactivity in thalamic nuclei, and regions with lesser immunoreactivity used gain set at 2 (Supplementary Fig. 6). Images were adjusted using linear brightness/contrast functions of Adobe Photoshop software. Identification of mouse lumbar spinal cord laminae followed Zeilhofer et al. (2012) and brain regions followed Franklin and Paxinos (1997).

Quantification of Gal_1_-mCherry-immunoreactive neuron profiles in 16 μm sections of lumbar L4 and L5 DRG from control unaxotomized animals (*n* = 3) and axotomized animals seven days after axotomy (*n* = 3, ipsilateral; Section 2.3) was as reported, with counting of 6–10 sections and at least 800 profiles per DRG (Holmes et al., 2008).

### 2.10 Statistical analysis

Statistical significance of real-time quantitative RT-PCR results (Section 2.6) and changes in somatic cell membrane fluorescence after galanin addition (Section 2.8) were judged by two-tailed Student's *t*-test, with *P* values < 0.05 considered significant. *P* values of < 0.05 and < 0.01 are indicated by one and two asterisks, respectively.

### 2.11 Nomenclature

With respect to nomenclature, the genes *GalR1* and *GalR2* (official symbols, Mouse Genome Informatics Database) encode the gene products Gal_1_ and Gal_2_ (http://www.guidetopharmacology.org/GRAC/FamilyDisplayForward?familyId=27), and for convenience transcripts are designated here as Gal_1_ and Gal_2_ mRNAs.

## 3 Results

### 3.1 Generation of GalR1-mCherry and GalR2-hrGFP knock-in mouse lines

**Supplementary Fig. 1:**
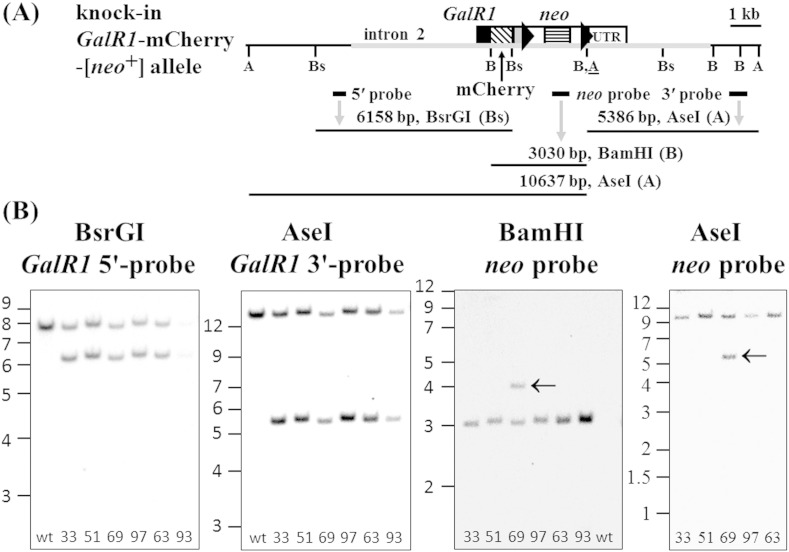
Southern blot analysis of ES cell clones validates correct insertion of the *GalR1*-mCherry-[*neo*^+^] targeting construct. (A) Schematic diagram of the knock-in *GalR1*-mCherry-[*neo*^+^] allele showing the relative locations of: the targeting construct (grey horizontal thickened line); *GalR1* exon 3 coding sequence (CDS; black filled box); mCherry CDS (diagonal banded box); the heterologous 3′-UTR (grey filled box); downstream FRT sites (right arrowheads) flanking an SV40-*neo* cassette selection marker; the endogenous 3′-UTR (UTR); restriction sites AseI (A), BsrGI (Bs), BamHI (B) and an introduced AseI restriction site immediately downstream of the 3′ FRT site (A); external 5′ and 3′ probes, *neo* probe, and hybridizing DNA fragments (see main article, Fig. 1A, for corresponding diagram of the endogenous allele). (B) DNA from six potential heterozygous *GalR1*-mCherry-[*neo*^+^] knock-in ES cell clones (33, 51, 63, 69, 93 and 97) and control wild-type mouse tail (wt) were digested and hybridized with: BsrGI and *GalR1* 5′ external probe (endogenous 7821 bp and knock-in 6158 bp; under-exposure for clone 93); AseI and *GalR1* 3′ external probe (endogenous 12,924 bp and knock-in 5386 bp); BamHI and *neo* probe (knock-in 3030 bp); or AseI and *neo* probe (knock-in 10,637 bp). The relative distance travelled by DNA ladder fragments (1 kb Plus, Life Technologies) are indicated in kb on the left of each panel. *Neo* was not detected in control wild-type mouse tail DNA, but an additional insertion of *neo*-hybridizing DNA was detected in ES cell clone 69 (arrowed) that was distinguishable from the correctly inserted knock-in fragment by digestion with BamHI or AseI. This additional *neo* insertion is due to only a fragment of introduced DNA (Fig. 1A, a grey horizontal thickened line), as the *neo*-hybridizing portion of AseI-digested DNA must be of at least 7366 bp which is larger than the additional hybridizing band of approximately 5500 bp, and quantitative genomic PCR detected only one copy of *GalR1*-mCherry.

**Supplementary Fig. 2:**
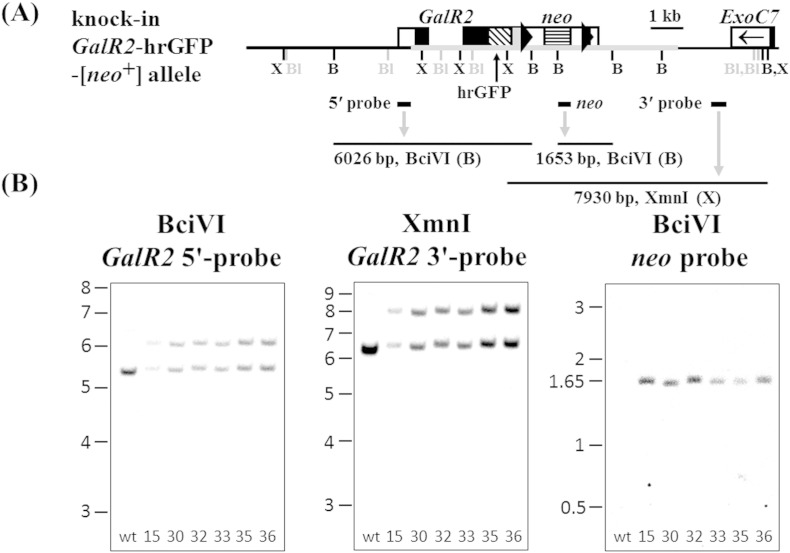
Southern blot analysis of ES cell clones validates correct insertion of the *GalR2*-hrGFP-[*neo*^+^] targeting construct. (A) Schematic diagram of the knock-in *GalR2*-hrGFP-[*neo*^+^] allele showing the relative locations of: the targeting construct (grey horizontal thickened line); the 3′ end of the *GalR2* CDS (exon 2, black filled box) joined to knock-in elements as in Supplementary Fig. 1A except for exchanging hrGFP (diagonal banded box) for mCherry; the downstream terminal exon of *ExoC7* on the other DNA strand (unfilled box, arrowhead); restriction sites XmnI (X), BlpI (Bl, greytone) and BciVI (B); and external 5′ and 3′ probes, *neo* probe, and hybridizing DNA fragments (see main article, Fig. 2A, for corresponding diagram of the endogenous allele). [BlpI sites are shown as used to characterize the [Δ*neo*] allele in main article Fig. 2.] (B) DNA from six potential heterozygous *GalR2*-hrGFP-[*neo*^+^] knock-in ES cell clones (15, 30, 32, 33, 35 and 36) and control wild-type mouse tail (wt) were digested and hybridized with: BciVI and *GalR2* 5′ external probe (*left panel*; endogenous 5366 bp and knock-in 6026 bp); XmnI and *GalR2* 3′ external probe (*middle panel*; endogenous 6222 bp and knock-in 7930 bp); and BciVI and *neo* probe (*right panel*; knock-in 1653 bp). DNA ladder as Fig. 1B.

**Supplementary Fig. 3:**
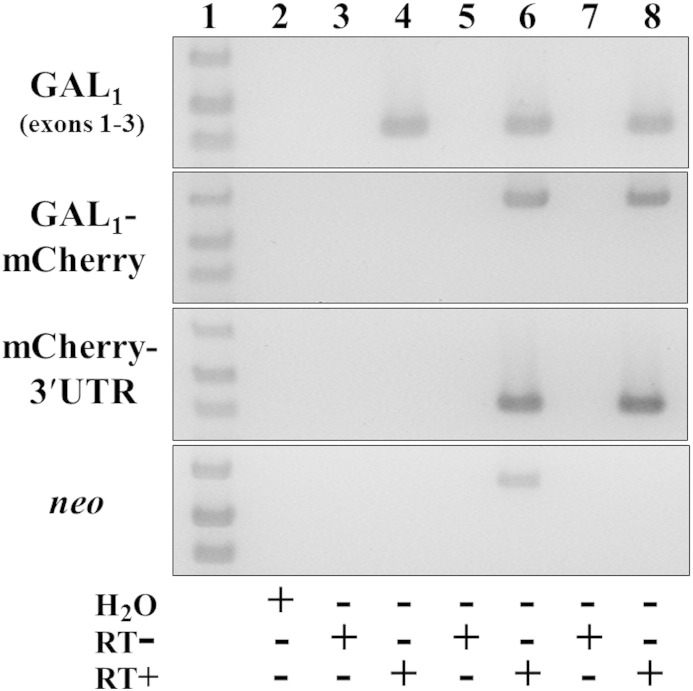
The knocked-in *GalR1*-mCherry gene is expressed and correctly spliced in adult DRG by RT-PCR analysis. Wild-type control (lanes 3 and 4), heterozygous *GalR1*-mCherry-[*neo*^+^] knock-in (lanes 5 and 6) and homozygous *GalR1*-mCherry-[Δ*neo*] knock-in mice (lanes 7 and 8) each expressed the correctly spliced product of *GalR1* exons 1–3 coding sequences (*top panel*, lanes 4, 6 and 8; 430 bp), whereas only knock-in mice express both the spliced product of *GalR1* exons 1–3 fused to mCherry (637 bp) and mCherry to the heterologous 3′-UTR (431 bp) (*middle panels*, lanes 6 and 8). Expression of *neo* was only detected in heterozygous *GalR1*-mCherry-[*neo*^+^] knock-in mice (*bottom panel*, lane 6; 630 bp). Knock-in mice were each of line 33. Lane 1 is a DNA ladder (1 kb Plus, Life Technologies) showing bands of 400, 500 and 650 bp; lane 2 is a water control; other even-numbered lanes are RT-PCRs using reverse transcribed RNA; and other odd-numbered lanes are corresponding reactions using RNA that has not been reverse transcribed (RT − controls), in which no products were detected.

**Supplementary Fig. 4:**
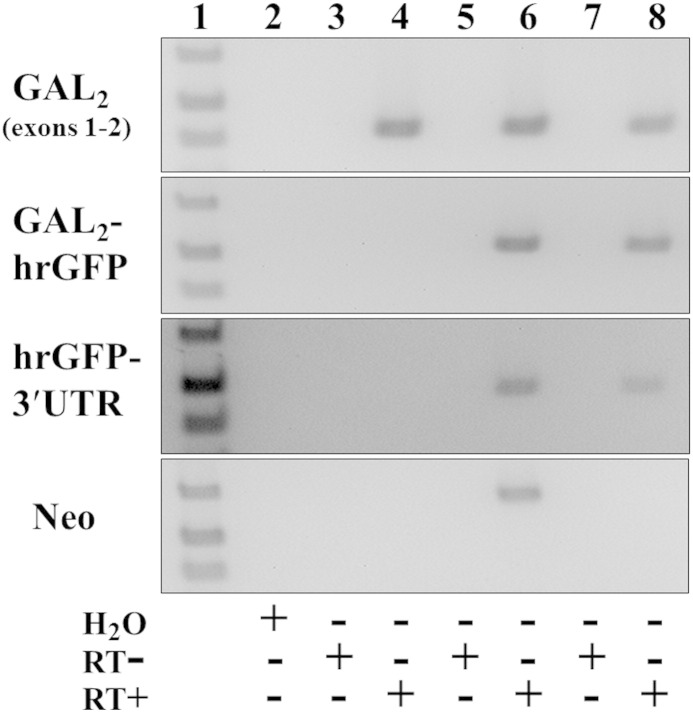
The knocked-in *GalR2*-hrGFP gene is expressed and correctly spliced in adult DRG by RT-PCR analysis. Wild-type control (lanes 3 and 4), heterozygous *GalR2*-hrGFP-[*neo*^+^] knock-in (lanes 5 and 6) and homozygous *GalR2*-hrGFP-[Δ*neo*] knock-in mice (lanes 7 and 8) each expressed the correctly spliced product of *GalR2* exons 1-2 coding sequences (*top panel*, lanes 4, 6 and 8; 426 bp), whereas only knock-in mice express both GalR2 (exon 2) fused to hrGFP (533 bp) and hrGFP to the heterologous 3′-UTR (483 bp) (*middle panels*, lanes 6 and 8). Expression of *neo* was only detected in heterozygous *GalR2*-hrGFP-[*neo*^+^] knock-in mice (*bottom panel*, lane 6; 630 bp). Knock-in mice were each of line 32. Lane 1 is a DNA ladder showing bands of 400, 500 and 650 bp (1 kb Plus, Life Technologies); lane 2 is a water control; other even-numbered lanes are RT-PCRs using reverse transcribed RNA; and other odd-numbered lanes are corresponding reactions using RNA that has not been reverse transcribed (RT − controls), in which no products were detected.

The coding sequences of the fluorescent tags mCherry or hrGFP were cloned in-frame onto the 3′ ends of genomic *GalR1* or *GalR2* coding sequences by lambda Red-mediated recombineering, and a smaller subcloned fragment of each was electroporated into embryonic stem (ES) cells (see Materials and methods section; Figs. 1A and 2A, *middle panels*). Screening of ES cell clones by real-time quantitative genomic PCR resulted in 6/97 *GalR1*-mCherry clones and 10/40 *GalR2*-hrGFP clones with one correctly targeted, knock-in allele and one endogenous allele of the receptor gene. Correct targeting of selected ES cell clones was confirmed further by Southern blot analysis using 5′ and 3′ probes external to the targeting construct sequence, and internal *neo* probe (Supplementary Figs. 1 and 2). Three heterozygous knock-in ES cell clones of *GalR1*-mCherry or *GalR2*-hrGFP were injected into blastocysts to generate chimeras, from which germline transmission of the knock-in allele was confirmed by PCR genotyping for all three *GalR1*-mCherry lines (33, 63 and 97) and one *GalR2*-hrGFP line (32). Expression of knock-in receptor and *neo* mRNAs in adult DRG was demonstrated by RT-PCR (Supplementary Figs. 3 and 4).

Gene expression can be perturbed by the presence of a nearby *neo* gene under the control of a strong promoter (Ema et al., 2006; Maguire et al., 2014; Revell et al., 2005), so the downstream SV40-*neo* cassette selection marker flanked by FRT (FLP recombination target) sites (Figs. 1A and 2A, *middle panels*) was excised in vivo by crossing with *ACTB*:*FLPe* transgenic mice in which FLPe recombinase is under the direction of the human β-actin promoter (Buchholz et al., 1998; Rodriguez et al., 2000). Animals heterozygous for *GalR1*-mCherry, negative for *neo*, and positive for deletion of the SV40-*neo* cassette (Δ*neo*) by PCR genotyping were inbred to produce homozygous *GalR1*-mCherry-[Δ*neo*] knock-in mice (lines 33 and 97), and the corresponding strategy was used to produce homozygous *GalR2*-hrGFP-[Δ*neo*] knock-in mice (see Materials and methods section). DNA sequencing of Δ*neo* PCR genotyping products confirmed that FLPe had precisely excised the SV40-*neo* cassette resulting in a product with a single remaining recombined FRT site (Figs. 1A and 2A, *bottom panels*), while Southern blot analysis confirmed both the expected different restriction fragment sizes of knock-in compared to endogenous alleles and the absence of *neo*-hybridizing sequences in *GalR1*-mCherry lines 33 (Fig. 1B) and 97 (data not shown), and in the *GalR2*-hrGFP knock-in (Fig. 2B).

### 3.2 mRNA expression in DRG from knock-in mice

Expression and correct splicing of transcripts from the knock-in genes was assessed by RT-PCR of adult DRG, the *GalR1* and *GalR2* genes having respectively three and two exons (Pang et al., 1998; Wang et al., 1997). *GalR1*-mCherry line 33 expressed the expected spliced product of *GalR1* exons 1–3 fused to mCherry coding sequence (637 bp), mCherry to the heterologous 3′-UTR (431 bp), and did not express *neo* (Supplementary Fig. 3). Similar results were obtained for *GalR1*-mCherry line 97 (data not shown) which was therefore discontinued. The *GalR2*-hrGFP knock-in expressed the spliced product of coding sequences from *GalR2* exons 1–2 (426 bp), both *GalR2* fused to hrGFP (533 bp) and hrGFP to the heterologous 3′-UTR (483 bp), and did not express *neo* (Supplementary Fig. 4). The identities of knock-in RT-PCR products were confirmed by DNA sequencing, and it is important to note that products that did not span exons were shown to be RT-dependent i.e. derived from mRNA and not genomic DNA contamination (Supplementary Figs. 3 and 4).

Quantitative RT-PCR was used to determine whether expression levels of the knock-in mRNA either differed from wild-type mouse endogenous receptor or led to adaptive regulation of galanin and galanin receptor mRNAs (Hawes et al., 2005; Hohmann et al., 2003). Expression levels in DRG from five individual wild-type, *GalR1*-mCherry knock-in or *GalR2*-hrGFP knock-in mice were compared using previously published assays for Gal_1_, Gal_2_ and galanin spliced coding sequences (Hobson et al., 2006) that are common to both the wild-type and knock-in animals. We have reported previously that Gal_3_ mRNA expression in mouse DRG is too low for reliable comparisons (Hobson et al., 2006). Compared to wild-type mice, there were no significant differences in the levels of Gal_1_ or galanin mRNAs detected in either *GalR1*-mCherry knock-in (0.856 ± 0.177, *P* = 0.165 and 0.904 ± 0.072, *P* = 0.477, respectively) or *GalR2*-hrGFP knock-in mice (1.261 ± 0.099, *P* = 0.067 and 1.102 ± 0.195, *P* = 0.850, respectively). In contrast, levels of Gal_2_ mRNA varied depending on genotype, with a small but significant increase in *GalR1*-mCherry knock-ins (1.361 ± 0.076, *P* = 0.020; *P < 0.05) and a significant decrease in *GalR2*-hrGFP knock-ins (0.524 ± 0.080, *P* = 0.004; **P < 0.01).

The quantitative RT-PCR results were also used to compare the relative abundance of different transcripts (Marsh et al., 2012; Pollema-Mays et al., 2013). In wild-type DRG Gal_1_ and Gal_2_ mRNAs were amplified to detectable levels (mean threshold cycle, C_t_) at respectively 25.37 and 32.58 cycles (each *n* = 5), the difference of 7.21 cycles corresponding to Gal_2_ being 148-fold less highly expressed than Gal_1_. These comparatively low expression levels of Gal_2_ mRNA were RT-dependent, i.e. derived from mRNA, as no products were detected in RT-minus controls at 50 cycles.

### 3.3 Live-cell imaging of Gal_1_-mCherry and Gal_2_-hrGFP proteins in primary DRG neurons from knock-in mice

**Fig. 3:**
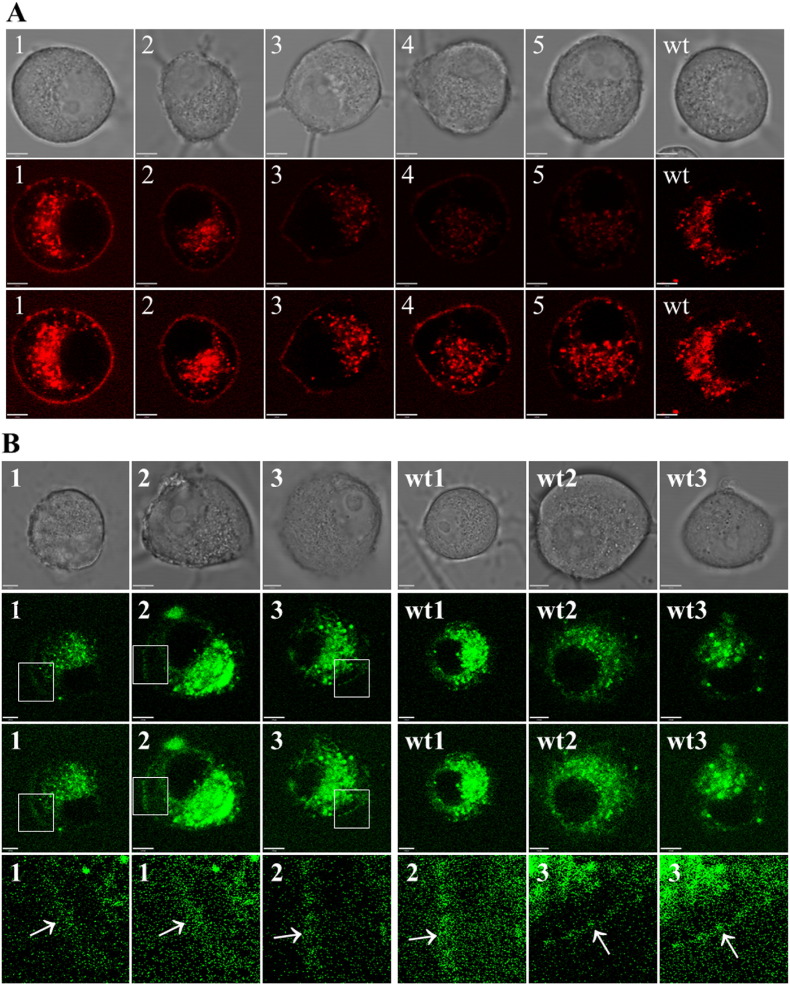
Detection of Gal_1_-mCherry and Gal_2_-hrGFP fluorescence by live-cell imaging. (A) Primary DRG neurons from *GalR1*-mCherry knock-in (cells 1–5) or wild-type (wt) mice are shown in brightfield images (*top row*); corresponding original fluorescent images, each acquired during the same imaging session with identical confocal settings (*middle row*); and corresponding adjusted images to emphasize somatic cell membrane fluorescence (see Materials and methods section), with cells 4, 5 and wt images treated identically (*bottom row*). Note that both knock-in and wild-type cells have *intracellular* autofluorescence within the neuronal cell body due to lipofuscin, but only knock-in cells have somatic *cell membrane* fluorescence. (B) Primary DRG neurons from *GalR2*-hrGFP knock-in (cells 1–3) or wild-type (wt1–3) mice are shown in brightfield images (*top row*); corresponding adjusted confocal fluorescent images (*second row*); corresponding brighter images to emphasize somatic cell membrane fluorescence (*third row*); and magnified fluorescent images from knock-in cells 1–3 at each of the two relative brightness levels (areas boxed in second and third rows), with arrows indicating the position of somatic cell membranes (*bottom row*). Knock-in and wild-type cells from the same imaging sessions are shown (1/wt1, 2/wt2 or 3/wt3). Note that expression of Gal_2_-hrGFP fluorescence at the somatic *cell membrane* is at the limits of detection, and that both knock-in and wild-type cells have *intracellular* autofluorescence within the cell body due to lipofuscin. For (A) and (B), each fluorescent image is a single optical section from a *z* stack, and the scale bar is 5 μm.

In preliminary experiments to detect mCherry or hrGFP fluorescence by confocal microscopy, the PC12 cell line was transiently transfected with either human (h) Gal_1_-mCherry or hGal_2_-hrGFP cDNAs under the control of the strong CMV (cytomegalovirus) promoter/enhancer (data not shown). Imaging conditions were then established for detecting the much lower fluorescence of each knock-in gene product under the control of the endogenous promoter in primary DRG neurons (Materials and methods section). Cells were detected that expressed a wide range of different levels of Gal_1_-mCherry fluorescence associated with the somatic cell membrane (Fig. 3A, *middle row*). However, under these sensitive detection conditions the globular, *intracellular* autofluorescence from lipofuscin (see Discussion section; Terman and Brunk, 2004) was also readily apparent within the cell bodies of both knock-in and wild-type neurons, which made it impossible to distinguish between specific Gal_1_-mCherry fluorescence and non-specific lipofuscin autofluorescence *within* the neuronal cell bodies (Schnell et al., 1999). Growth cones were also examined, as lipofuscin is limited to the neuronal cell body (Gorenstein and Ribak, 1985), but Gal_1_-mCherry fluorescence was not detected.

Gal_2_-hrGFP fluorescence was expected to be more difficult to detect than Gal_1_-mCherry, due to the much lower mRNA expression (see above, Section 3.2). Gal_2_-hrGFP fluorescence at the somatic cell membrane was near the limits of detection, but was detectable in 3 of 150 DRG neurons analysed (Fig. 3B).

**Fig. 4:**
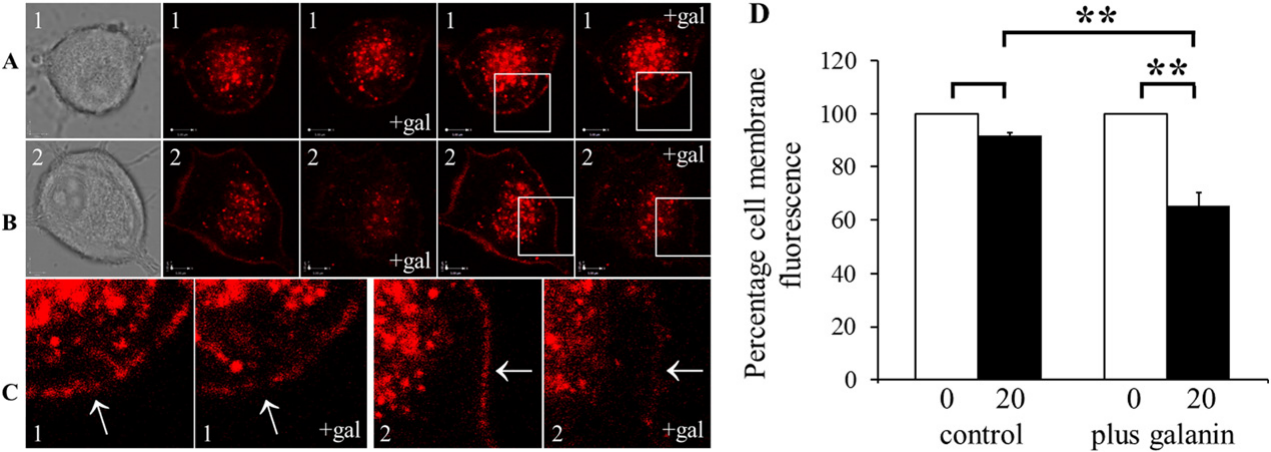
Live-cell imaging of changes in Gal_1_-mCherry fluorescence at the somatic cell membrane following the addition of galanin. (A) Primary DRG neuron ‘1’ from *GalR1*-mCherry knock-in mouse shown (left to right) in brightfield image; corresponding original confocal fluorescent images at time zero and at 20 min after addition of 1 μM galanin (+ gal), from which quantifications in (D) were made; and the same fluorescent images adjusted to emphasize cell membrane fluorescence (see Materials and methods section). (B) Corresponding images of primary DRG neuron ‘2’. (C) Magnified fluorescent images of each cell at both timepoints (areas boxed in (A) and (B)), with arrows indicating the position of somatic cell membranes. Each fluorescent image is a single optical section, and the scale bar ((A) and (B)) is 5 μm. (D) Quantification of changes in Gal_1_-mCherry fluorescence at the somatic cell membrane. Compared to somatic cell membrane fluorescence at time zero, defined as 100%, there was not a significant decrease in somatic cell membrane fluorescence in control neurons re-imaging 20 min later (*left*; 91.75 ± 3.76%, *n* = 4; *P* = 0.1155). In other cells imaged and then re-imaged 20 min after 1 μM galanin addition, there was a significant decrease in somatic cell membrane fluorescence to 65.17 ± 5.18% (*right*; *n* = 4; *P* = 0.0067; **, *P* < 0.01). The somatic cell membrane fluorescence at the 20 min timepoint also is significantly decreased in galanin-treated compared to control neurons (*n* = 4; *P* = 0.0089; **, *P* < 0.01).

A previous study in transfected CHO cells demonstrated a decrease in fluorescently-tagged Gal_1_ receptor at the cell membrane following incubation with 100 nM–2 μM galanin (Wirz et al., 2005). To study the effect of galanin on Gal_1_-mCherry localization in primary DRG neurons, cells with comparatively high somatic cell membrane fluorescence were selected and imaged at 37 °C in a recently available medium with reduced background fluorescence (Materials and methods section). In control Gal_1_-mCherry neurons there was no significant decrease in somatic cell membrane fluorescence on re-imaging at 20 min, i.e. no apparent photo-bleaching, whereas the somatic cell membrane fluorescence significantly decreased by 35% in neurons re-imaged 20 min after the addition of 1 μM galanin (65.17 ± 5.18%; *P* = 0.0067; Fig. 4), demonstrating agonist-induced internalization.

### 3.4 Immunohistochemical detection of Gal_1_-mCherry protein in DRG, spinal cord and brain of adult knock-in mice

**Fig. 5:**
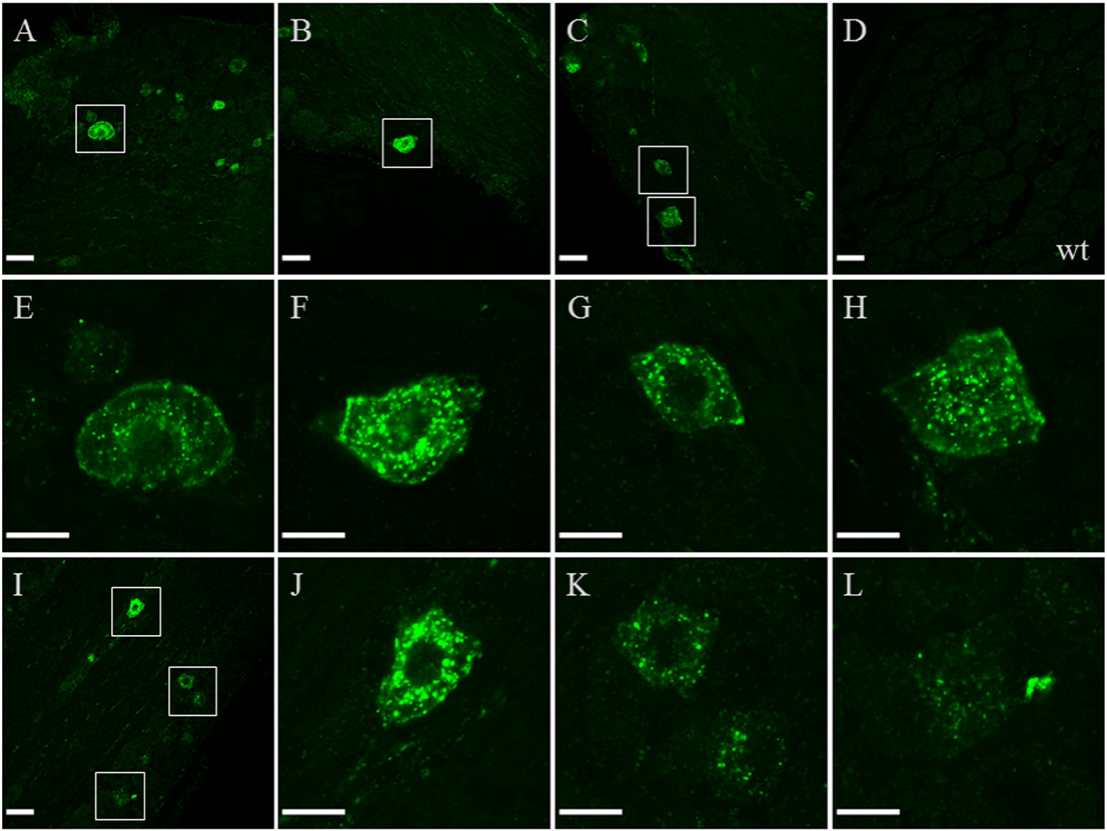
Detection of Gal_1_-mCherry immunofluorescence in DRG. (A–C) Three confocal images of adult *GalR1*-mCherry knock-in lumbar DRG show some neurons with comparatively high immunofluorescence (green colour, boxed) and others with lesser immunofluorescence whereas, (D) in adult wild-type (wt) DRG specific immunofluorescence is not detected (40 × objective, scale bar 40 μm). (E–H) Higher magnification images of highly immunofluorescent neurons (boxed in (A-C)) show localization of Gal_1_-mCherry to the somatic cell membrane and a punctate distribution within the cytoplasm (40 × objective, zoom 6 ×; scale bar 15 μm). (I) In a confocal image with high, moderate and low level immunofluorescent cells (boxed; scale bar 40 μm), at higher magnification somatic cell membrane localization is apparent in the high expressing neuron (J) but not in either a moderate (K, top) or two low expressing neurons (K bottom and L; scale bar 15 μm). Each fluorescent image is a single optical section from a *z* stack.

In order to detect Gal_1_-mCherry protein expression with higher sensitivity, we used a previously characterized antibody to mCherry (Erbs et al., 2015; Voigt et al., 2012) that specifically detected the protein expressed from CMV-driven hGal_1_-mCherry cDNA in transiently transfected PC12 cells (data not shown). Gal_1_-mCherry immunofluorescence in adult lumbar DRG was detected as comparatively high expression in a restricted subset of neurons (Fig. 5A–C, I), with localization to the somatic cell membrane and a punctate distribution within the cytoplasm (Fig. 5E–H, J), whereas in neurons with lesser immunofluorescence localization to the cell surface was often not detected (Fig. 5K–L). In wild-type DRG specific immunofluorescence was not apparent (Fig. 5D). Previously we reported quantitative RT-PCR results showing that Gal_1_ mRNA decreased by 37% in wild-type mouse DRG seven days after sciatic nerve transection (axotomy; Hobson et al., 2006), whereas here by cell counts of Gal_1_-mCherry immunoreactive neuronal profiles no apparent difference was detected between 10.9 ± 1.9% in control lumbar L4 and L5 DRG and 9.5 ± 1.1% in ipsilateral L4 and L5 DRG seven days after axotomy (each *n* = 3, ± SEM).

**Supplementary Fig. 5:**
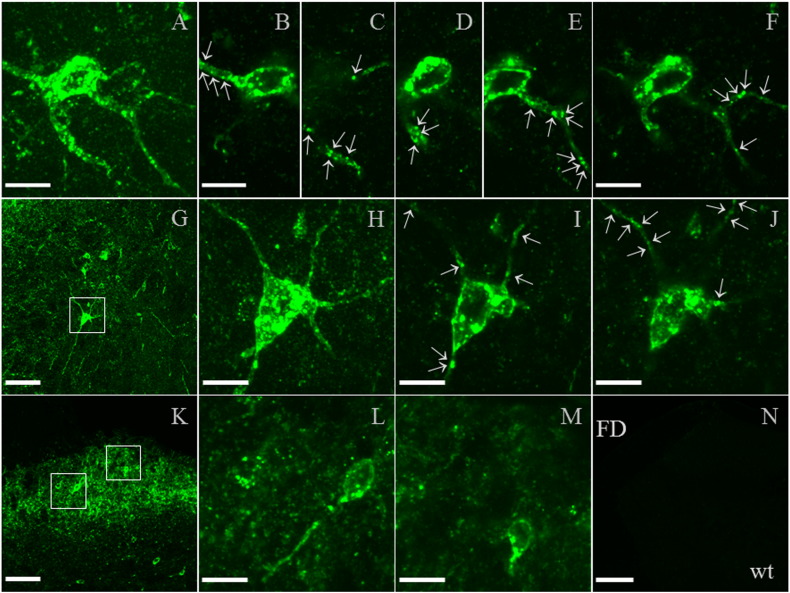
Additional confocal images of Gal_1_-mCherry immunofluorescence in adult spinal cord. (A–F) Additional images of the multipolar neuron from the lamina III/IV border area shown in the main article (Fig. 6J). The multipolar morphology of the neuron is shown by a merged image of 20 *z*-sections (A; 40 × objective, zoom 6 ×), and single *z*-sections at different planes show multiple, discrete Gal_1_-mCherry immunofluorescent puncta located within the neuronal processes (arrows, B–F). (G–J) The morphology of another multipolar neuron from the lamina III/IV border area is shown by merged confocal sections at lower magnification (G, boxed; 40 × objective; 12 *z*-sections); and higher magnification (H; 40 × objective, zoom 6 ×; 15 *z*-sections), while single *z*-sections show multiple, discrete Gal_1_-mCherry immunofluorescent puncta located within the neuronal processes (arrows, I–J). (K–M) Additional examples of intrinsic neurons within lamina I–II are shown (K, boxed; 40 × objective), that at higher magnification show localization of Gal_1_-mCherry immunofluorescence to the somatic cell membrane (L–M; 40 × objective, zoom 6 ×; L, 5 *z*-sections). (N) Wild-type (wt) dorsal horn lacks specific immunofluorescence (20 × objective; scale bar 150 μm; FD, funiculus dorsalis) at the same brightness level as used for imaging Gal_1_-mCherry knock-in tissue in the main article (Fig. 6B–D and N). Each fluorescent image is a single optical section from a *z* stack, unless indicated. Scale bars are: 15 μm for 40 × objective, zoom 6 ×; 70 μm for 40 × objective; and 150 μm for 20 × objective images.

**Fig. 6:**
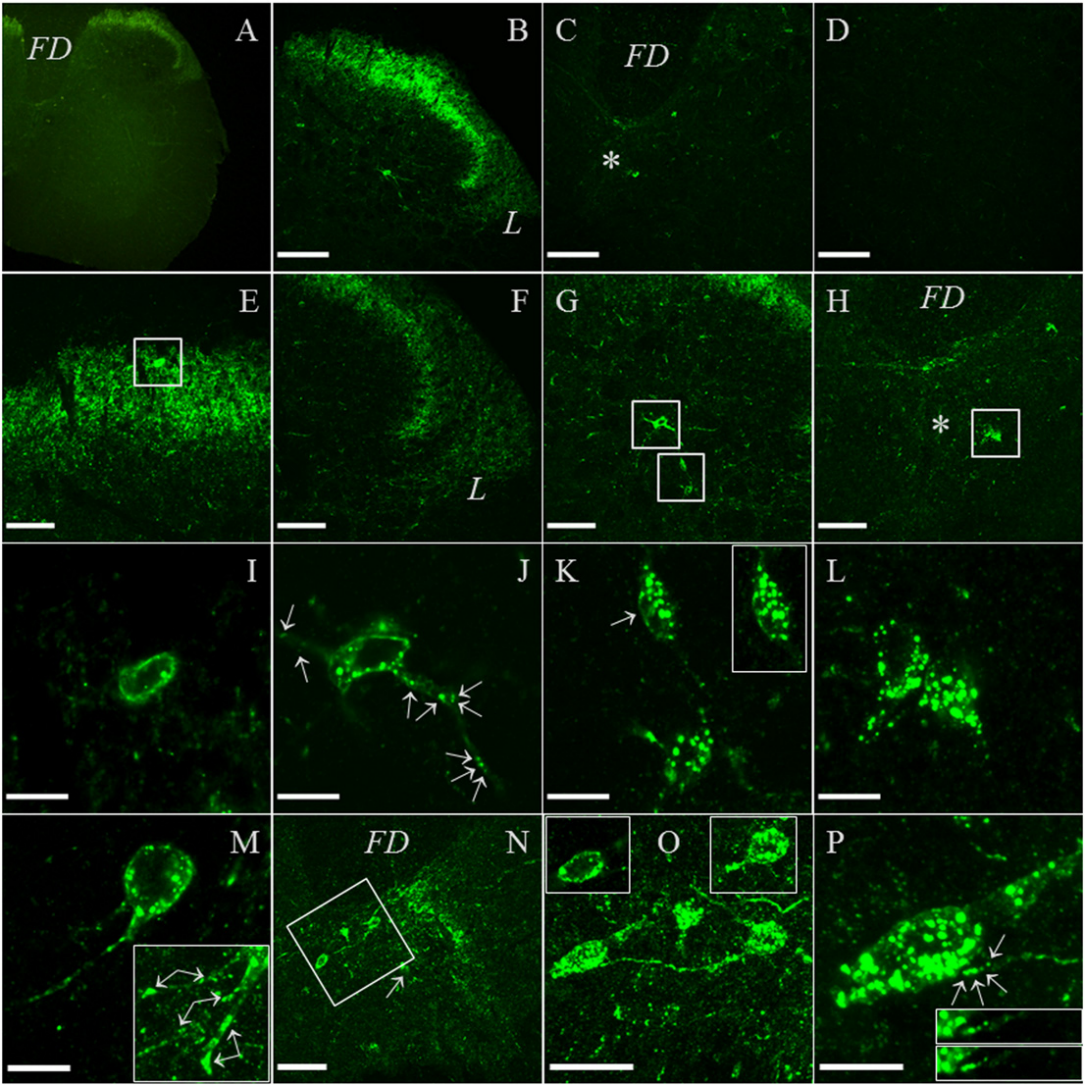
Detection of Gal_1_-mCherry immunofluorescence in spinal cord. (A) Low-power photomicrograph (10 × objective) and (B–D) confocal images (20 × objective) of adult *GalR1*-mCherry knock-in lumbar spinal cord showing high levels of immunofluorescence in the superficial dorsal horn laminae I–II, with lesser levels in the lateral spinal nucleus (LSN, labelled ‘*L*’) and around the central canal (white asterisk) in lamina X (A–C), whereas little immunofluorescence was detected in the ventral horn (D). (E–H) Higher magnified confocal images (40 × objective) of mid superficial dorsal horn (E); lateral superficial dorsal horn and LSN (labelled ‘*L*’; F); lamina III/IV border (G); and lamina X (H; central canal, white asterisk); with examples of local cell bodies boxed. (I–L, M) Higher magnification confocal images (40 × objective, zoom 6 ×) of local neurons (boxed in row above) show distinct somatic cell membrane localization in highly fluorescent cells of the superficial dorsal horn (I), lamina III/IV border (J) and lamina X (M; location arrowed in N, mainly different plane), whereas in less fluorescent neurons cell surface localization may (K, arrow; insert merged 3 *z*-sections) or may not be detected (K–L; L, merged 4 *z*-sections). Note the multiple Gal_1_-mCherry immunofluorescent puncta within neuronal processes (J, arrows; additional images in Supplementary Fig. 5A–F), and insert of M shows three main neuronal projections (each double arrows; merged 16 *z*-sections, 1 μm intervals). (N–P) Confocal images of medial spinal cord including a lamina IV neuron sending a process laterally to another Gal_1_-mCherry expressing neuron (boxed, N; 40 × objective); as shown at higher magnification to show the neuronal process (O; objective 40 ×, zoom 3 ×; merged 8 *z*-sections, 1 μm intervals), with insert images showing somatic cell membrane localization of each cell (right cell, merged 3 *z*-sections); and at further magnification Gal_1_-mCherry puncta are seen in the terminal process of the lamina IV neuron (arrows, P; objective 40 ×, zoom 8 ×; merged 7 *z*-sections, 1 μm intervals), with insert images of details from successive *z*-sections of the terminal process. *FD* is funiculus dorsalis. Each confocal image is a single optical section from a *z* stack unless indicated, with (B–D) at same brightness intensity, as are (E–H and N). Scale bars: 150 μm for 20 × objective; 70 μm for 40 × objective; 40 μm for 40 × objective with zoom 3 ×; and 15 μm for 40 × objective with either zoom 6 × or 8 ×.

Within adult lumbar spinal cord, high levels of Gal_1_-mCherry immunofluorescence were detected in the superficial layers of the dorsal horn in lamina I–II processes, with lesser levels in the lateral spinal nucleus (LSN) and around the central canal in lamina X (Fig. 6A–C). Little immunofluorescence was detected in the ventral horn (Fig. 6D), and specific immunofluorescence was not detected in wild-type cord (Supplementary Fig. 5). Occasional intrinsic cell bodies were detected in lamina I/II, the lamina III/IV border and lamina X (boxed, Fig. 6E, G and H; Supplementary Fig. 5), the most highly expressing cells showing somatic cell membrane localization and puncta within the cell bodies (Fig. 6I–M). Outward transport of Gal_1_-mCherry from the cell body was detected as puncta within cell processes of multipolar neurons within the lamina III/IV border area (Fig. 6J, with further sections in Supplementary Fig. 5–J). In addition, we show a neuron within the medial part of lamina IV (Zeilhofer et al., 2012) extending a process laterally ~ 75 μm to end in a terminal containing multiple Gal_1_-mCherry immunofluorescent puncta, apposed to another Gal_1_-mCherry expressing neuron (Fig. 6N–P).

**Fig. 7:**
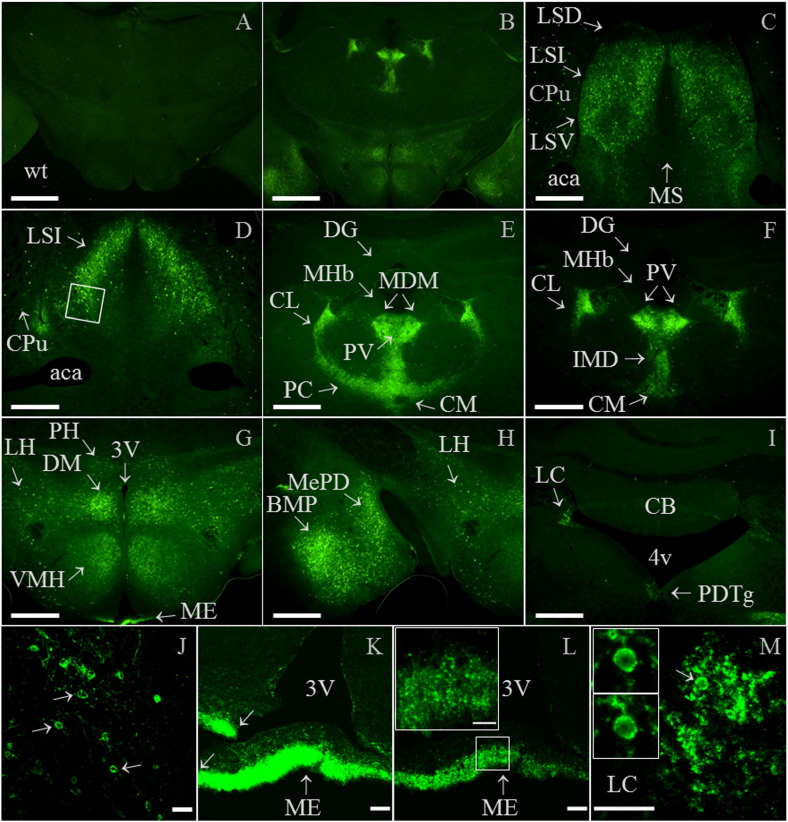
Detection of Gal_1_-mCherry immunofluorescence in brain. (A–B) In low-power micrographs (2.5 ×), specific immunofluorescence is not detected in adult wild-type (wt) brain (A), whereas in a similar section of adult *GalR1*-mCherry knock-in brain, high levels of Gal_1_-mCherry immunoreactivity were detected (B; see F-H for same section at higher magnifications). (C–I) Higher magnification images (5 ×) show high immunoreactivity in: (C) intermediate and ventral parts of lateral septal nuclei (LSI, LSV) and scattered cells within caudate putamen (CPu), but not in the dorsal part of the lateral septal nucleus (LSD) at this level or in medial septal nucleus (MS); (D) LSI, and scattered cells within CPu; (E–F) paraventricular-, medial mediodorsal-, central medial-, paracentral-, central lateral- and intermediodorsal-thalamic nuclei (PV; MDM; CM; PC; CL; IMD), but not in dentate gyrus (DG) or medial habenula (MHb); (G) dorsomedial and ventromedial hypothalamic nuclei (DM; VMH), median eminence (ME; see below) and scattered cells within posterior and lateral hypothalamic areas (PH; LH); (H) posterior basomedial and posterodorsal medial amygdala nuclei (BMP; MePD) and LH; (I) locus coeruleus (LC) and posterodorsal tegmental nucleus (PDTg), but not cerebellum (CB). (J–M) Confocal images at higher magnification (J–L: 40 ×; M: 40 ×, zoom 3 ×; scale bars 40 μm) showing: (J) somatic cell membrane localization in some neurons within the intermediate part of the lateral septal nucleus (LSI; arrows; area boxed in (D)); (K) high levels of expression in median eminence (ME) shown either over-bright to show location of third ventricle (3V; left pair of arrows showing join of torn tissue; 30 *z*-sections) or less-bright image (L; 3 *z*-sections), with insert of boxed area magnified to show immunoreactive fibres and an absence of immunoreactive cell bodies (40 ×, zoom 6 ×; scale bar 15 μm; 3 *z*-sections); and (M) fibres within the locus coeruleus (LC) area, together with a single cell body (arrow; 20 *z*-sections), with inserts showing somatic cell membrane localization (single *z*-sections). Other abbreviations: aca, anterior part of anterior commissure; and 4V, 4th ventricle. Images (A–B) and (C–I) are each at the same brightness intensity, with scale bars of 1 mm (A–B, 2.5 × objective) or 0.5 mm (C–I, 5 × objective lens). Sections are similar to mouse brain atlas Figures 46 (A, B), 23 (C), 29 (D, J), 40 (E), 46 (F–H, K, L) and 79 (I, M) of Franklin and Paxinos (1997).

In wild-type brain no specific immunofluorescence was detected, whereas in similar sections of knock-in brain high levels of Gal_1_-mCherry immunoreactivity were detected within thalamus, hypothalamus and amygdala (Fig. 7A and B). Examples of the most immunoreactive brain regions are shown at higher magnification (5 ×), including the intermediate and ventral parts of the lateral septal nucleus (Fig. 7C and D); a number of thalamic nuclei including the paraventricular thalamic nucleus (Fig. 7E and F); the dorsomedial and ventromedial hypothalamic nuclei and median eminence (Fig. 7G); medial amygdala nuclei (Fig. 7H); and the locus coeruleus (Fig. 7I). Confocal images at higher magnification show localization of Gal_1_-mCherry to the somatic cell membrane of neurons within the lateral septal nucleus (Fig. 7J), high levels of expression in nerve endings within the external zone of the median eminence (Fig. 7K and L) and numerous nerve fibres in the locus coeruleus area (Fig. 7M).

**Supplementary Fig. 6:**
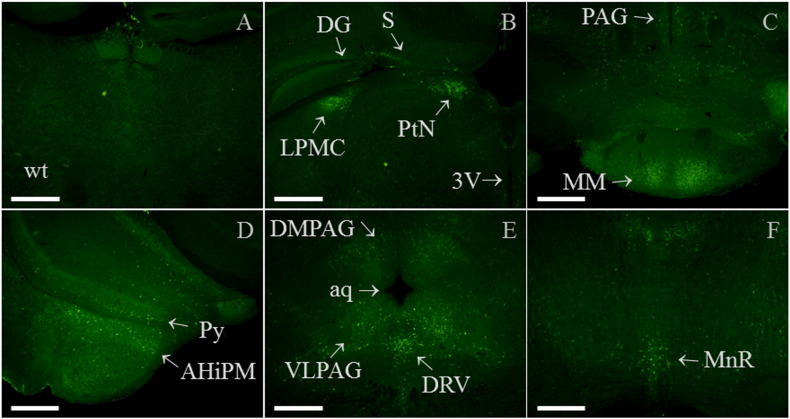
Photomicrograph images of Gal_1_-mCherry immunofluorescence in brain regions with lesser immunoreactivity. (A) In brighter images than main article Fig. 7 (see below) there is still a lack of specific immunofluorescence in adult wild-type (wt) thalamus, in a section similar to knock-in brain with high Gal_1_-mCherry immunoreactivity (Fig. 7F). Gal_1_-mCherry immunofluorescence is now more easily detected in (B): the mediocaudal part of the lateral posterior thalamic nucleus (LPMC), pretectal nucleus (PtN; medial or posterior parts, MPT/PPT) and subiculum (S), but not in dentate gyrus (DG); (C): medial mammillary nucleus (MM) and periaqueductal grey (PAG); (D): posteromedial hippocampal amygdala (AHiPM) and scattered cells in the ventral hippocampal pyramidal cell layer (Py); (E): dorsomedial and ventrolateral periaqueductal grey (DMPAG, VLPAG) and ventral part of dorsal raphe nucleus (DRV); and (F): median raphe nucleus (MnR). Other abbreviations are: aq, aqueduct and 3V, 3rd ventricle. Image brightness in main article Fig. 7 was constrained by the high immunoreactivity in thalamic nuclei, whereas here imaging used a ‘gain’ value of 2 rather than 1 with Image Manager IM50 software (Leica; Holmes et al., 2008). All images are micrographs using 5 × objective lens (scale bar 0.5 mm), (B)–(D) are from the same brain section (see Supplementary Fig. 7L) as are (E) and (F) (see Supplementary Fig. 7N and P). Sections are similar to mouse brain atlas Figures 46 (A), 54/55 (B–D) and 68 (E, F) of Franklin and Paxinos (1997).

**Supplementary Fig. 7:**
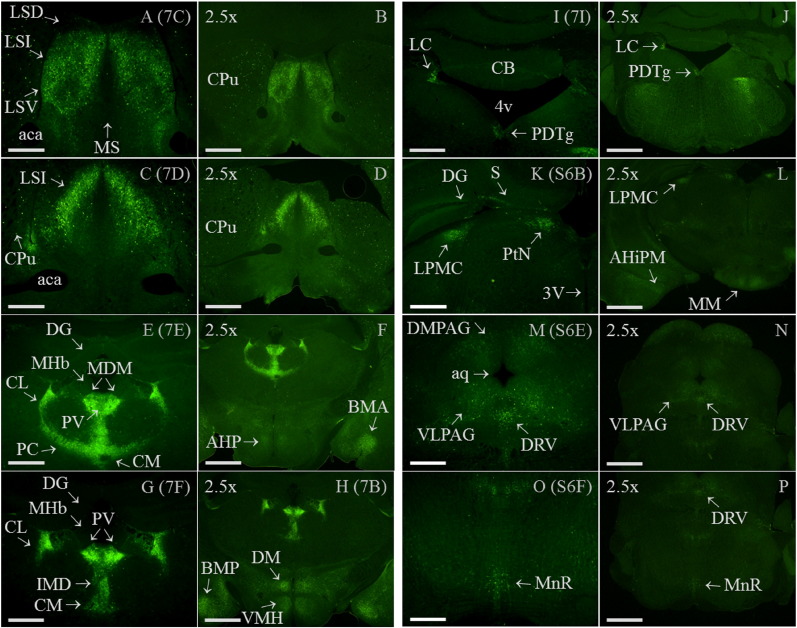
Comparison of high (5 ×) to low (2.5 ×) magnification photomicrograph images of Gal_1_-mCherry immunofluorescence in brain. Lefthand column is high power magnification images (5 ×) from main article Fig. 7C–F and I, and Supplementary Fig. 6B, E and F (designations shown in brackets, top right of each image). Righthand column is corresponding low power magnification images (designated ‘2.5 ×’ in top left of each image). Additional brain areas specified here are the anterior hypothalamic area (AHP) and anterior part of basomedial amygdala (BMA) (Supplementary Fig. 7F). Note that 2.5 × magnification images of Supplementary Figs. 7H and L, respectively, also include the 5 × magnification images shown in Fig. 7G–H and Supplementary Fig. 6C–D. Scale bars are 0.5 mm (5 × objective) and 1 mm (2.5 × objective). Other abbreviations in alphabetical order are: aca, anterior part of anterior commissure; AHiPM, posteromedial hippocampal amygdala; aq, aqueduct; BMP, posterior part of basomedial amygdala nucleus; CB, cerebellum; CL, central lateral thalamic nucleus; CM, central medial thalamic nucleus; CPu, caudate putamen; DG, dentate gyrus; DM, dorsomedial ventromedial hypothalamic nucleus; DMPAG, dorsomedial part of periaqueductal grey; DRV, ventral part of dorsal raphe nucleus; IMD, intermediodorsal thalamic nucleus; LC, locus coeruleus; LPMC, lateral posterior thalamic nucleus; LSD, dorsal part of lateral septal nucleus; LSI, intermediate part of lateral septal nucleus; LSV, ventral part of lateral septal nucleus; MDM, medial part of mediodorsal thalamic nucleus; MHb, medial habenula; MM, medial mammillary nucleus; MnR, median raphe nucleus; MS, medial septal nucleus; PC, paracentral thalamic nucleus; PDTg, posterodorsal tegmental nucleus; PtN, pretectal nucleus; PV, paraventricular thalamic nucleus; S, subiculum; VLPAG, ventrolateral part of periaqueductal grey; VMH, ventromedial hypothalamic nucleus; 3V, 3rd ventricle; 4V, 4th ventricle.

Examples of brain regions with lesser Gal_1_-mCherry immunoreactivity (Materials and Methods) are shown in Supplementary Fig. 6. These include the mediocaudal part of the lateral posterior thalamic nucleus; pretectal nucleus; subiculum; medial mammillary nucleus; periaqueductal grey; posteromedial hippocampal amygdala; scattered cells in the ventral-most hippocampal pyramidal cell layer; the ventral part of the dorsal raphe nucleus and medial raphe nucleus. The correspondence of the higher magnification images (Fig. 7 and Supplementary Fig. 6) to those at a lower magnification are shown in Supplementary Fig. 7, which also localize Gal_1_-mCherry immunoreactivity in the anterior hypothalamic area and anterior part of basomedial amygdala (Supplementary Fig. 7).

### 3.5 Absence of immunohistochemical detection of Gal_2_-hrGFP protein in adult knock-in mouse tissues

A previously characterized antibody to hrGFP (Sakata et al., 2009; Zhang et al., 2013) specifically detected the protein expressed by CMV-driven hGal_2_-hrGFP cDNA in transiently transfected PC12 cells (data not shown). However, we could not detect specific Gal_2_-hrGFP protein expression by immunohistochemistry in adult normal DRG, spinal cord or brain, or in DRG and spinal cord after sciatic nerve axotomy which markedly induces Gal_2_ mRNA in rat ventral horn motoneurons (Brumovsky et al., 2006b). Immunofluorescence did not differ from wild-type tissue using a range of primary antibody dilutions (Materials and Methods), and omission of the primary antibody eliminated fluorescence. The inability to detect Gal_2_-hrGFP is compatible with the low endogenous expression of Gal_2_/Gal_2_-hrGFP mRNA (Section 3.2; see Discussion section).

## 4 Discussion

The goal of this study was to produce knock-in mice expressing fluorescently-tagged Gal_1_ or Gal_2_ receptors for live-cell functional imaging and immunohistochemical localization studies. The Gal_1_-mCherry and Gal_2_-hrGFP mRNAs were each expressed and correctly spliced in adult DRG from the respective knock-in mice, but the steady-state levels of Gal_2_-hrGFP mRNA were decreased by ~ 50% compared to the endogenous Gal_2_ transcript of wild-type DRG. This may be due to the presence of mRNA stabilization sequence(s) present in the endogenous Gal_2_ mRNA 3′-UTR that do not occur in the heterologous 3′-UTR of Gal_2_-hrGFP mRNA (Fig. 2A), such as the stabilization motif CCTnCCTG-like sequence ACTACCTG (Cohen et al., 2014; NM_010254).

The live-cell imaging of Gal_1_-mCherry and Gal_2_-hrGFP proteins was restricted to the somatic cell membrane, axons and growth cones due to lipofuscin autofluorescence *within* the soma (see below). This still allowed the detection of Gal_1_-mCherry fluorescence localized to the somatic cell membrane, plus the demonstration of galanin-dependent internalization in DRG neurons. Previously, [^125^I]-galanin binding to neuronal cell bodies of intact monkey DRG or human nodose ganglion (Sweerts et al., 2000; Zhang et al., 1995b), lacked the spatial resolution to distinguish cell membrane from intracellular binding, whereas the increased membrane excitability upon addition of galanin to acutely dissociated rat DRG neurons indicated functional receptor(s) localized to the somatic cell membrane (Kerekes et al., 2003). Intact DRG neuron cell bodies have been shown to express surface receptors for a variety of neuroactive substances e.g. the GPCRs neuropeptide Y (NPY) type Y1 receptor (Y1-R) and somatostatin type 2A receptor (Sstr2A) (Hanani, 2005; Shi et al., 2014; Zhang et al., 1994).

Lipofuscin autofluorescence is known to complicate fluorescent microscopy of brain, spinal cord and DRG (Schnell et al., 1999), for example identifying GFP-GR (glucocorticoid receptor) knock-in expression in brain (Usuku et al., 2005), and will be exaggerated by the very sensitive detection conditions used here (Spitzer et al., 2011; Materials and methods section). Lipofuscin is composed of oxidized protein and lipid degradation residues, that is located within lysosomes but cannot be degraded by lysosomal hydrolases and so accumulates over time within post-mitotic cells such as neurons or cardiac myocytes (Sulzer et al., 2008; Terman and Brunk, 2004). Studies on lipofuscin generally focus on aged animals, but it has been reported in 2 month old rat brain and heart (Ikeda et al., 1985; Nakano et al., 1995; Sulzer et al., 2008), and in 6 week old brain and 1–3 month old spinal cord from mouse (Bandyopadhyay et al., 2014; Constantinides et al., 1986; Usuku et al., 2005). The classical defining characteristic of lipofuscin is broad spectrum autofluorescence (Eldred et al., 1982; Sulzer et al., 2008), as we detected in 500–700 nm emission spectral scans of wild-type DRG neurons (data not shown), which overlaps the commonly used fluorophores such as mCherry and hrGFP with emission peaks of respectively 610 and 506 nm (Shaner et al., 2004; Stratagene-Agilent).

Gal_1_-mCherry protein was localized within DRG, spinal cord and brain using the higher sensitivity of immunohistochemistry (Figs. 5-7). Similarly, in the δ-opioid receptor (DOR)-EGFP knock-in mouse fluorescence was often weak and required EGFP-specific antibodies for proper visualization (Erbs et al., 2015), and DOR and Gal_1_ mRNA are expressed at similar levels in mouse DRG based on next generation RNA sequencing (RNA-Seq; Supplementary Table 1 of Thakur et al., 2014). Cellular localization of a specific GPCR can differ between different regions of the nervous system, for example DOR-EGFP is localized to the somatic cell membrane in DRG but is not readily detected at the cell surface of spinal cord neurons (Ceredig and Massotte, 2014; Erbs et al., 2015; Nathanson, 2008). In highly immunofluorescent Gal_1_-mCherry neurons, somatic cell membrane localization was detected in DRG (Fig. 5E–H, J), in spinal cord lamina I/II, lamina III/IV border, medial lamina IV and lamina X (Fig. 6I–K, M and O; Supplementary Fig. 5) and within brain in the lateral septal nucleus and locus coeruleus (Fig. 7J and M). In the live-cell imaging of primary DRG neurons we did not detect Gal_1_-mCherry fluorescence in growth cones or axons, which could be clearly viewed because lipofuscin autofluorescence is limited to the neuronal cell body (Gorenstein and Ribak, 1985). In contrast, the combination of the immunohistochemistry and the generally restricted expression among intrinsic spinal cord neurons allowed the detection of Gal_1_-mCherry transport within cell processes as puncta in successive confocal images of neurons within the lamina III/IV border area (Fig. 6J and Supplementary Fig. 5–J) and medial lamina IV (Fig. 6N–P; Zeilhofer et al., 2012).

Previously, we detected Gal_1_ mRNA in mouse DRG by RT-PCR (Hobson et al., 2006), and here detected Gal_1_-mCherry immunofluorescence in a restricted subset of neurons (Fig. 5A–C, I) corresponding to 10.9% of lumbar L4 and L5 neuronal profiles. Published data on Gal_1_ mRNA + neuron profiles is not available for mouse DRG, but in adult rat DRG the initial figure was 23% whereas in a more sensitive study using labelled riboprobes the figure was 51% (Kerekes et al., 2003; O'Donnell et al., 1999; Xu et al., 1996). Differences between mouse and rat DRG neuron profiles are not uncommon, for example between 7 and 20% for NPY receptor Y1 mRNA + or between 39 and 8% for P2X5 immunopositive profiles (Shi et al., 1998; Zeng et al., 2013). In addition, immunohistochemical analysis can give lower numbers of positive neurons compared to ISH, as for example with DOR-EGFP immunostaining compared to DOR mRNA ISH in mouse DRG (Scherrer et al., 2009; Wang et al., 2010). The apparent lack of effect of axotomy on Gal_1_-mCherry immunoreactive neuronal profiles (9.5% versus control 10.9%), in combination with our previous quantitative RT-PCR results showing a 37% decrease in Gal_1_ mRNA in wild-type DRG (Hobson et al., 2006), may be explained in part by a threshold for immunoreactive detection. If the mRNA decrease occurs mainly in the highly expressing neurons they would still maintain sufficient expression to be counted as Gal_1_-mCherry immunopositive. Consistent with this, an ISH study of NPY mRNA expression in rat superior cervical ganglion (SCG) after axotomy detected a 40% decrease in average grain density/neuron, but the number of NPY mRNA + neurons was unchanged (Kroesen et al., 1997).

In DRG the potential sources of galanin to bind to somatic cell membrane Gal_1_ are either via fenestrated capillaries that allow access into the neuronal extracellular space (Hanani, 2005) or locally produced ligand. Basal expression of galanin mRNA and protein in DRG are low but increase dramatically after axotomy (Villar et al., 1989; Zhang et al., 1995a), when it is abundant in the Golgi region and also present stored in large dense-core vesicles within the cell body (Zhang et al., 1995a). Somatic exocytosis of galanin has yet to be studied, though it has been demonstrated for substance P (SP) and calcitonin gene-related peptide (CGRP) (Liu et al., 2011; Trueta and De-Miguel, 2012) and references therein), and in addition to post-axotomy would also be relevant following galanin induction in several other neuropathic pain models (Lang et al., 2015) and during nerve regeneration following nerve crush injury (Villar et al., 1989).

In mouse spinal cord expression of Gal_1_ mRNA has previously been detected by northern blot and RT-PCR (Jacoby et al., 2002; Wang et al., 1997), and by ISH was reported as enriched in the dorsal horn (Table 1 of Guo et al., 2012). We detected dense Gal_1_-mCherry immunofluorescence in the superficial dorsal horn laminae I–II with lesser levels in the lateral spinal nucleus and lamina X, similar to previous rat Gal_1_ mRNA ISH studies (Brumovsky et al., 2006b; O'Donnell et al., 1999). It will be of interest to determine the expression of other neurochemical markers within intrinsic neurons highly expressing Gal_1_-mCherry. For example, the potential relationship of the multipolar neurons of the lamina III/IV border (Fig. 6G and J; Supplementary Fig. 5) to the occasional multipolar neurons of similar location that express galanin, NPY receptor Y1-R or somatostatin receptor Sstr2 (Brumovsky et al., 2006a; Melander et al., 1986; Shi et al., 2014), and the relationship of neurons lateral to the central canal and potentially interacting medial lamina IV neurons (Fig. 6M–P) to galanin or Y1R expressing cells (Brumovsky et al., 2006a; Ch'ng et al., 1985; Melander et al., 1986).

The heterogeneous distribution of Gal_1_ mRNA in mouse forebrain has been determined by ISH (Hohmann et al., 2003) and was highly similar to [^125^I]-galanin binding sites, which were not detected in mice deficient for Gal_1_ (Jungnickel and Gundlach, 2005). The examples of brain regions shown as the most Gal_1_-mCherry immunoreactive (Fig. 7 and Supplementary Fig. 7) correspond, with three exceptions, to areas expressing Gal_1_ mRNA which on an intensity scale of + to ++++ (from weak to very dense) were in the range of ++ to ++++ (Hohmann et al., 2003). The ISH study did not extend caudally to the locus coeruleus and posterodorsal tegmental nucleus (Fig. 7I) which are known to bind [^125^I]-galanin (Jungnickel and Gundlach, 2005), so the apparent discrepancy is in median eminence (ME) between the high level of Gal_1_-mCherry immunofluorescence (Fig. 7G, K and L) and the absence of Gal_1_ mRNA expression in both mouse and rat (Fig. 4d of Hohmann et al., 2003; Mitchell et al., 1997). However, Gal_1_ mRNA is detected in most hypothalamic nuclei that project towards it, and as there is a high density of [^125^I]-galanin binding sites in the ME of mouse and rat (Jungnickel and Gundlach, 2005), these findings are therefore compatible with the hypothesis that median eminence Gal_1_ receptors are transported and play a local role at the nerve terminals (Mitchell et al., 1997). This is now clearly demonstrated by Gal_1_-mCherry immunofluorescence localized to densely packed nerve endings within the external zone of the ME (Fig. 7K–L), a blood–brain barrier-free circumventricular organ (Fekete and Lechan, 2014; Mullier et al., 2010) with a very high density of galanin-immunoreactive fibres in both mouse and rat (Melander et al., 1986; Perez et al., 2001).

Brain regions with lesser Gal_1_-mCherry immunoreactivity (Materials and methods section; Supplementary Fig. 6) correspond to Gal_1_ mRNA intensities in the range + to ++ (Hohmann et al., 2003), or in the case of the lateral posterior thalamic nucleus is known to bind [^125^I]-galanin (Jungnickel and Gundlach, 2005), while the dorsal raphe nucleus (DR) and median raphe nucleus were too caudal to be included in the ISH study. Gal_1_ mRNA is expressed in adult rat DR (Burazin et al., 2000; O'Donnell et al., 1999), but on the basis of a lack of Gal_1_-immunoreactivity or detection of Gal_1_ mRNA by ISH in mouse, a species difference in expression was proposed (Larm et al., 2003). Since then, [^125^I]-galanin binding sites and weak expression of Gal_1_ mRNA in the ventral part of mouse DR have been detected (Borroto-Escuela et al., 2010; Jungnickel and Gundlach, 2005). Here we show Gal_1_-mCherry immunoreactivity in the DR (Supplementary Fig. 6), galanin receptor agonists having implicated mouse DR Gal_1_ in facilitating limbic seizures (Mazarati et al., 2005).

The difficulty in detecting Gal_2_-hrGFP fluorescence in the somatic cell membrane of DRG neurons can mainly be ascribed to comparatively low mRNA expression from the endogenous promoter. In wild-type mouse DRG the endogenous Gal_2_ mRNA was 148-fold less highly expressed than Gal_1_ (Section 3.2), which is consistent with data used in our previous report (Hobson et al., 2006) in which Gal_2_ was 136-fold less highly expressed (difference of 7.09 cycles; each *n* = 5). This wide difference in expression of the two receptors is confirmed by RNA-Seq data of adult mouse DRG in which Gal_1_ and Gal_2_ had FPKM (Fragment Per Kilobase of exon per Million fragments mapped) values of 4.865 and 0.020, respectively (Supplementary Table 1 of Thakur et al., 2014). The expression levels of Gal_2_-hrGFP in DRG, spinal cord and brain may be below the sensitivity for detection by immunohistochemistry, but Gal_2_ expression appears to be sufficient to affect behaviour. Gal_2_-deficient mice have anxiogenic-like and depression-like phenotypes (Bailey et al., 2007; Lu et al., 2008), and compounds with a marked preference for binding Gal_2_ over Gal_1_ have anticonvulsive and anti-nociceptive activities (Metcalf et al., 2015; Robertson et al., 2010).

**Fig. 8:**
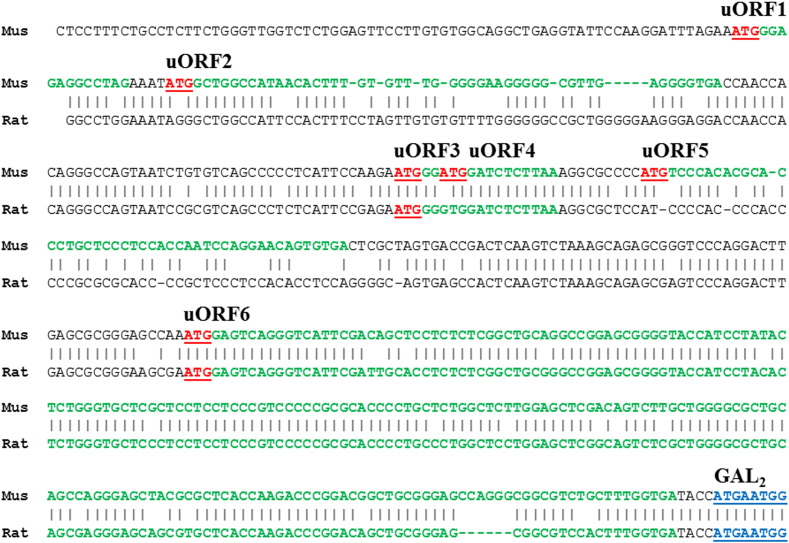
Upstream open reading frames (uORFs) are present in mouse and rat Gal_2_ mRNA. Alignment of the 5′-UTR sequences of mouse and rat Gal_2_ mRNA showing uORFs, with ATG triplets (*bold underlined*, *red*) and associated ORFs (*bold*, *green*) upstream of the Gal_2_ coding region (‘Gal_2_’; *bold*, *blue*) indicated, and note that of the six uORFs in the mouse sequence (uORF1-6) there is conservation of uORF3 and uORF6 in rat. For clarity, the open reading frame of mouse uORF4 is not indicated. Sequences shown are mouse (Mus) and rat mRNA reference sequences (respectively NM_010254 with 5′-UTR of 547 nt, and NM_019172 with 5′-UTR of only 20 nt), with additional rat 5′-UTR sequence derived from expressed sequence tag (EST) CB780446 and further extended by EST CB713787 to 466 nt. The rat sequence shares identity with the predicted rat Gal_2_ mRNA sequence (XM_008768432) and rat hypothalamic cDNA clones including 5′-UTRs of 492 and 296 bp have been reported, though the sequences are not available (Howard et al., 1997; U94322). The mouse sequence is supported by cDNAs/ESTs including uORF1-6 (AK053776, BC116980, BB607282), uORF2-6 (BC116982) and uORF5-6 (AK132050, BY712835) isolated from embryonic day 12 embryo or head, neonatal day 0 eyeball, and brain.

Of direct relevance to the very low levels of Gal_2_-hrGFP, is the observation that Gal_2_ is likely to be under translational control mediated by upstream open reading frames (uORFs) within the 5′-UTR. This will tend to diminish translation of the main ORF by reducing the number of ribosomes reaching and initiating at the main start codon (Barbosa et al., 2013; Calvo et al., 2009). Unlike the 5′-UTR of mouse and rat Gal_1_ mRNA sequences that do not contain upstream ATG triplets (NM_008082; NM_012958), those of mouse Gal_2_ contains six uORFs (here designated uORF1–6) whilst rat Gal_2_ contains only two uORFs (Fig. 8). These latter two are conserved between mouse and rat (hereafter uORF3 and uORF6) in both relative position and approximate length, potentially encoding 5 and 71/69 amino acids respectively, and the mouse/rat uORF6 terminates only 4 nucleotides upstream of the Gal_2_ initiation codon. Such proximity has previously been correlated with translational repression of the main coding sequence (Child et al., 1999; Kozak, 1987; but see Calvo et al., 2009), as has uORF length (Calvo et al., 2009; Kozak, 2001), and uORF-mediated repression may or may not depend on the potential uORF-encoded protein sequence (Child et al., 1999; Iacono et al., 2005; Morris and Geballe, 2000) but conservation of the uORF mRNA sequence between species suggests a functional selection (Churbanov et al., 2005; Crowe et al., 2006; Iacono et al., 2005).

Ribosome profiling (ribo-seq) data based on deep-sequencing of ribosome-protected mRNA fragments is not yet available for mouse neurons (Michel et al., 2014), but is for initiating ribosomes of the mouse ES cell line E14 in which Gal_2_ has low expression and ribosome density (Ingolia et al., 2011). The predicted initiation site of uORF6 has a ribosome footprint, with a peak density ~ 1.7-fold higher than at the Gal_2_ initiation site (Materials and methods section, Section 2.1; Ingolia et al., 2011; Michel et al., 2014), which suggests a functional role for the conserved uORF6. Intriguingly, endoplasmic reticulum (ER)-stress and a number of other stressors that transiently inhibit the translation of most mRNAs can also promote translation initiation of mRNAs with uORFs (Barbosa et al., 2013; Spriggs et al., 2010), and ER-stress is known to occur in vivo following neuronal axon damage, spinal cord injury or brain trauma (Li et al., 2013; Nakka et al., in press; Yasuda et al., 2014). However, attempts to detect Gal_2_-hrGFP protein in DRG or spinal cord at 3 or 7 days after sciatic nerve peripheral axotomy were unsuccessful (Section 3.5). Further studies could be useful either in DRG after carrageenan-induced inflammation when Gal_2_ mRNA is induced in rat (Sten Shi et al., 1997) or in the carotid body which has 100-fold more Gal_2_ than Gal_1_ mRNA in rat (Hawes and Picciotto, 2005; Porzionato et al., 2010). There is now a clear need for in vitro studies on the mechanism of Gal_2_ translational control using various stressors (Barbosa et al., 2013; Spriggs et al., 2010) in order to understand both the conditions and time-courses of Gal_2_ protein expression, preferably in a neuronal-like cell line.

## 5 Conclusions

The generation and initial characterization of the Gal_1_-mCherry knock-in mice will allow more detailed regional tissue distribution studies, co-localization studies, interactome analysis (Bauch et al., 2014) and provide an impetus to understand the function of Gal_1_ at the somatic cell membrane. Expression of Gal_2_-hrGFP was at the limits of detection and a possible mechanism involving uORFs is discussed.

The following are the supplementary data related to this article.

Supplementary material

Supplementary data to this article can be found online at http://dx.doi.org/10.1016/j.mcn.2015.08.006.

## Competing interests

The authors declare that they have no competing financial interests.
