# Supplementary material for: The generation of knock-in mice expressing fluorescently tagged galanin receptors 1 and 2
Source: Mol Cell Neurosci. 2015 Sep;68:258–71. doi: 10.1016/j.mcn.2015.08.006 (PMC4604734; doi:10.1016/j.mcn.2015.08.006)
Supplement: Supplementary file 1 — Supplementary material [file mmc1.docx]

**Supplemental Data**

**The generation of knock-in mice expressing fluorescently tagged galanin receptors 1 and 2**.

**Niall Kerr, Fiona E. Holmes, Sally-Ann Hobson, Penny Vanderplank, Alan Leard, Nina Balthasar and David Wynick**

**Supplementary Materials and Methods**

*Real-time quantitative genomic PCR of ES cell clones*

Relative gene copy number of ES cell clone DNA (Geneta) was determined by real-time quantitative (TaqMan) PCR assays, using the comparative threshold cycle (C_t_) method (Liu *et al*, 2003; Hobson *et al*, 2006). Primer and probe sets were designed using default parameters of Primer Express software (Applied Biosystems) or followed the previously described apolipoprotein B (ApoB) set (Liu et al., 2003). All probes had a 5' fluorescent reporter dye FAM (6-carboxy-fluorescein) and the 3' quencher dye TAMRA (6-carboxy-tetramethyl-rhodamine), and all primers and probe sets were synthesized by Applied Biosystems.

For each potential heterozygous *GalR1*-mCherry knock-in ES cell clone, three assays were set up in separate wells of the same 96 well reaction plate: (1) endogenous *GalR1* detected with forward primer 5'-GAAAACAAGAGCCGGATGGA-3' (R1cdsF), reverse primer 5'-CACACATGGGAGCTGGAAGTC-3' (R1utrR) and non-extendable probe 5'-CACGTGGGTGCAGTTGGTGGATG-3' (R1cdsTAQ); (2) *GalR1*-mCherry allele detected with forward primer R1cdsF, reverse primer 5'-AGCGCATGAACTCCTTGATGA-3' (mCherryR) and probe R1cdsTAQ; and (3) the homozygous endogenous control *ApoB* (Liu et al., 2003). For each potential heterozygous *GalR2*-hrGFP knock-in clone the assays were: (1) endogenous *GalR2* detected with primers 5'-ACCCGCACTTCCCAACTG-3' (R2cdsF) and 5'-CTGTTAGATGCCCTTTGGTCCTT-3' (R2utrR), and probe 5'-ACAGGCTGGATCGAGGGTTCTACTCAAGGT-3' (R2cdsTAQ); (2) *GalR2*-hrGFP allele detected with forward primer R2cdsF and 5'-CAGGATCTGCTTGCTCACCAT-3' (GfpR), and probe R2cdsTAQ; and (3) ApoB. Different primer concentrations and amounts of DNA were tested to define PCR conditions, and replicate 25 μl PCRs were set up as previously reported (Hobson et al., 2006).

*Southern blots of ES cell clones and tissue*

10 μg of ES cell clone DNA (Geneta) or mouse tail DNA was digested with restriction enzyme (New England Biolabs) for 6 hours, separated on a 0.8 % agarose gel and transferred to Hybond-N+ membrane (GE Healthcare).

External probes for *GalR1* and *GalR2* were PCR-amplified from bMQ mouse BACs (Source BioScience) using primers 5'-CTCCCCAAGATGCAACTCAAAGGAG-3' and 5'-CACATTTTCGAATGGATACAGCACAAG-3' for *GalR1* 5' probe (440 basepairs, bp; intron 2); 5'-cagtcGACATGTAGACTTCAGGAGCAGACTG-3' and 5'-gtgtcgACACTCTCATAGGCAACACTGGGT-3 (flanked by SalI restriction sites, underlined) for *GalR1* 3' probe (591 bp, uncloned extended 3'-UTR; Wang et al., 1997; NM_008082); 5'-CTTCTGGGTTGGTCTCTGGAGTTC-3' and 5'-CAGCCGAGAGAGGAGCTGTCGA-3' for *GalR2* 5' probe (352 bp, 5'-UTR); and 5'-GCTGAACCAGAACACAAAGCCATC-3' and 5'-CTGGAGCCATCGTCAGTACAGCA-3' for *GalR2* 3' probe (503 bp, downstream from gene). *Neo* probe (405 bp) was amplified from vector hrGFP-FRT*neo*FRT (see above) using primers 5'-GCATACGCTTGATCCGGCTACCT-3' and 5'-CGATAGAAGGCGATGCGCTGCGAA-3'. PCR products were each TA-cloned into vector pCRII-TOPO (Life Technologies) and DNA sequenced.

Probes were synthesized by random primer labelling (Life Technologies) of 50 ng DNA insert with [α-^32^P]-dCTP (Perkin Elmer), and purified through a G-50 microcolumn (GE Healthcare). Blot radioactivity was detected on a Storm 860 PhosphorImager and analysed using ImageQuant software (Molecular Dynamics).

*Detection of knock-in mRNA expression in DRG by RT-PCR*

DRG from individual, adult mice were frozen on dry ice and stored at -80 ^o^C. Total RNA isolation, reactions containing reverse transcriptase (RT+) or without enzyme (RT- control; (Kerr et al., 2004), and RT-PCR cycling conditions were as reported (Kerr et al., 2010), except for RT-PCR annealing at 64 ^o^C unless otherwise stated. Primer pairs used were 5'-GTCTTTTCCATCGGGACAGCAACCA-3' (R1Fmu) and 5'-TCGAACTCGTGGCCGTTCACGGA-3' for Gal_1_ (exons 1-3) to mCherry; 5'-cggcgtggtgaccgtgaccca-3' and 5'-CGAGGTCGACGGTATCGATAAGCT-3' for mCherry to heterologous 3'-UTR (GC-rich); primer R1Fmu and 5'-CACTTGAACACTTGCTTGTACGCCT-3' for total Gal_1_ (Gal_1_ and Gal_1_-mCherry, exons 1-3); 5'-CACTACCTCTGGCGCACAGTTGAC-3' and 5'-GTGTTCTTCAGGATCTGCTTGCTCA-3' for Gal_2_ (exon 2) to hrGFP (62 ^o^C annealing); 5'-GAAGGACTTCCCCGAGTACCACT-3' and 5'-TCAGGGGGAGGTGTGGGAGGTT-3' for hrGFP to heterologous 3'-UTR (GC-rich; 62 ^o^C annealing); 5'-CGATTGGGTGTTTGGCTCACTGCT-3' and 5'-CTTGGCGCGCTGGGAACCTGAG-3' for total Gal_2_ (Gal_2_ and Gal_2_-hrGFP, exons 1-2; GC-rich; 62 ^o^C annealing); and 5'-GCATACGCTTGATCCGGCTACCT-3' and 5'-CTCCTTCCGTGTTTCAGTTAGCCT-3' (herpes simplex virus (HSV) thymidine kinase (*TK*) 3'-UTR; McKnight, 1980) for *neo*. ‘GC-rich’ refers to the use of GC-rich solution (Roche) in the RT-PCR reactions.

**Supplemental references**

Franklin, K.B.J., Paxinos, G., 1997. The Mouse Brain in Stereotaxic Coordinates. Academic Press, San Diego.

Hobson, S.A., Holmes, F.E., Kerr, N.C.H., Pope, R.J., Wynick, D., 2006. Mice deficient for galanin receptor 2 have decreased neurite outgrowth from adult sensory neurons and impaired pain-like behaviour. J.Neurochem. 99, 1000-1010.

Holmes, F.E., Arnott, N., Vanderplank, P., Kerr, N.C., Longbrake, E.E., Popovich, P.G., Imai, T., Combadiere, C., Murphy, P.M., Wynick, D., 2008. Intra-neural administration of fractalkine attenuates neuropathic pain-related behaviour. Journal of Neurochemistry 106, 640-649.

Kerr, N., Pintzas, A., Holmes, F., Hobson, S.A., Pope, R., Wallace, M., Wasylyk, C., Wasylyk, B., Wynick, D., 2010. The expression of ELK transcription factors in adult DRG: Novel isoforms, antisense transcripts and upregulation by nerve damage. Molecular & Cellular Neurosciences 44, 165-177.

Kerr, N.C.H., Holmes, F.E., Wynick, D., 2004. Novel isoforms of the sodium channels Nav1.8 and Nav1.5 are produced by a conserved mechanism in mouse and rat. Journal of Biological Chemistry 279, 24826-24833.

Liu, D.P., Schmidt, C., Billings, T., Davisson, M.T., 2003. Quantitative PCR genotyping assay for the Ts65Dn mouse model of Down syndrome. Biotechniques 35, 1170-1174, 1176, 1178.

McKnight, S.L., 1980. The nucleotide sequence and transcript map of the herpes simplex virus thymidine kinase gene. Nucleic Acids Res 8, 5949-5964.

Wang, S., He, C., Maguire, M.T., Clemmons, A.L., Burrier, R.E., Guzzi, M.F., Strader, C.D., Parker, E.M., Bayne, M.L., 1997. Genomic organization and functional characterization of the mouse GalR1 galanin receptor. FEBS Lett 411, 225-230.
